# Applying the Bradford Hill Criteria for Causation to Repetitive Head Impacts and Chronic Traumatic Encephalopathy

**DOI:** 10.3389/fneur.2022.938163

**Published:** 2022-07-22

**Authors:** Christopher J. Nowinski, Samantha C. Bureau, Michael E. Buckland, Maurice A. Curtis, Daniel H. Daneshvar, Richard L. M. Faull, Lea T. Grinberg, Elisa L. Hill-Yardin, Helen C. Murray, Alan J. Pearce, Catherine M. Suter, Adam J. White, Adam M. Finkel, Robert C. Cantu

**Affiliations:** ^1^Concussion Legacy Foundation, Boston, MA, United States; ^2^Department of Neuropathology, Royal Prince Alfred Hospital, Camperdown, NSW, Australia; ^3^School of Medical Sciences, University of Sydney, Camperdown, NSW, Australia; ^4^Department of Anatomy and Medical Imaging and Centre for Brain Research, Faculty of Medical and Health Science, University of Auckland, Auckland, New Zealand; ^5^Department of Physical Medicine and Rehabilitation, Harvard Medical School, Boston, MA, United States; ^6^Department of Physical Medicine and Rehabilitation, Massachusetts General Hospital, Boston, MA, United States; ^7^Department of Physical Medicine and Rehabilitation, Spaulding Rehabilitation Hospital, Boston, MA, United States; ^8^Memory and Aging Center, Department of Neurology, University of California, San Francisco, San Francisco, CA, United States; ^9^Global Brain Health Institute, University of California, San Francisco, San Francisco, CA, United States; ^10^Department of Pathology, University of Sao Paulo Medical School, São Paulo, Brazil; ^11^Department of Pathology, University of California, San Francisco, San Francisco, CA, United States; ^12^School of Health and Biomedical Sciences, STEM College, RMIT University, Bundoora, VIC, Australia; ^13^Department of Anatomy & Physiology, The University of Melbourne, Parkville, VIC, Australia; ^14^College of Science, Health, and Engineering, La Trobe University, Melbourne, VIC, Australia; ^15^Department of Sport, Health Science, and Social Work, Oxford Brookes University, Oxford, United Kingdom; ^16^Concussion Legacy Foundation UK, Cheltenham, United Kingdom; ^17^Department of Environmental Health Sciences, University of Michigan School of Public Health, Ann Arbor, MI, United States; ^18^Department of Neurology, Boston University School of Medicine, Boston, MA, United States; ^19^Department of Neurosurgery, Emerson Hospital, Concord, MA, United States

**Keywords:** chronic traumatic encephalopathy (CTE), repetitive head impact, Bradford Hill, causation, punch drunk, public health, epidemiology

## Abstract

Chronic traumatic encephalopathy (CTE) is a neurodegenerative disease associated with a history of repetitive head impacts (RHI). CTE was described in boxers as early as the 1920s and by the 1950s it was widely accepted that hits to the head caused some boxers to become “punch drunk.” However, the recent discovery of CTE in American and Australian-rules football, soccer, rugby, ice hockey, and other sports has resulted in renewed debate on whether the relationship between RHI and CTE is causal. Identifying the strength of the evidential relationship between CTE and RHI has implications for public health and medico-legal issues. From a public health perspective, environmentally caused diseases can be mitigated or prevented. Medico-legally, millions of children are exposed to RHI through sports participation; this demographic is too young to legally consent to any potential long-term risks associated with this exposure. To better understand the strength of evidence underlying the possible causal relationship between RHI and CTE, we examined the medical literature through the Bradford Hill criteria for causation. The Bradford Hill criteria, first proposed in 1965 by Sir Austin Bradford Hill, provide a framework to determine if one can justifiably move from an observed association to a verdict of causation. The Bradford Hill criteria include nine viewpoints by which to evaluate human epidemiologic evidence to determine if causation can be deduced: strength, consistency, specificity, temporality, biological gradient, plausibility, coherence, experiment, and analogy. We explored the question of causation by evaluating studies on CTE as it relates to RHI exposure. Through this lens, we found convincing evidence of a causal relationship between RHI and CTE, as well as an absence of evidence-based alternative explanations. By organizing the CTE literature through this framework, we hope to advance the global conversation on CTE mitigation efforts.

## Introduction

Repetitive head impacts (RHI), defined as the cumulative exposure to recurrent concussive and subconcussive events, have long been associated with the neurodegenerative disease chronic traumatic encephalopathy (CTE). Dr. Harrison Martland is credited with first identifying the syndrome that was later called CTE in his article *Punch Drunk*, published in the *Journal of the American Medical Association* in 1928. Martland found evidence of traumatic brain injuries in the post-mortem brains of boxers and proposed a clinical syndrome based on his experience treating retired boxers (1). Pathologically, Martland described multiple “ring hemorrhages” with perivascular distribution in the deeper structures of the brain.

Over the following decades, case reports in the literature proposed more features of the neuropathology and clinical syndrome (2). Pathological findings included case reports published in 1973 by Corsellis et al. from 15 retired boxers with “a characteristic pattern of cerebral change” they proposed “underlie many features of the punch-drunk syndrome,” which represented the largest CTE case series identified prior to 2013 (3). Corsellis et al. described neurofibrillary tangle (NFT) pathology, ventricular dilatation, and the presence of a cavum septum pellucidum (CSP) as features of CTE. In 1990, Roberts et al. (4) identified the first case of CTE from intimate partner violence in a 76-year-old woman, noting NFT pathology in the frontal cortex, an absence of senile plaques, and a large CSP. In 1992, Hof et al. (5) noted that NFTs accumulate in neocortical layers II and III in dementia pugilistica, and in 1996, Geddes et al. (6) described an abnormal pattern of perivascular NFT accumulation in the frontal lobe of a 23-year-old boxer they attributed to RHI.

In the 20th century, a causal relationship was widely accepted by the public and punch drunk boxers became common characters in literature (7), television (8), and film (9). In the 1954 film *On the Waterfront*, which won the 1955 Best Picture from the Academy of Motion Picture Arts and Sciences, Marlon Brando won the Academy Award for Best Actor by portraying a punch drunk boxer exhibiting many of the symptoms associated with CTE today.

However, in recent years, some have begun to publicly question whether the relationship between RHI and CTE is truly one of causation. The modern RHI/CTE debate began with the diagnosis of CTE in an American football player in 2005 (10). Prior to this case study, there appears to be no record of organizations like the National Football League (NFL), National Hockey League (NHL), or National Collegiate Athletic Association (NCAA) taking a public position against RHI/CTE causation. After 2005, all three leagues, and many others, went on record denying a link between RHI and CTE (11–14).

In 2013, the internationally influential Concussion in Sport Group (CISG) first addressed the question of causality in their consensus statement on concussions, writing, “It was further agreed that a cause and effect relationship has not as yet been demonstrated between CTE and concussions or exposure to contact sports” (15). In the next consensus statement update in 2017 they remained unconvinced, writing, “To date, a cause and effect relationship between CTE and concussions or exposure to contact sports has not been established” (16).

The CISG statements stand in contrast to the position of the US Centers for Disease Control and Prevention (CDC), which published a fact sheet in 2019 that states, “Most research suggests that CTE is caused in part by exposure to repeated traumatic brain injuries, including concussions, and repeated hits to the head, called subconcussive head impacts (i.e., head impacts that do not cause symptoms of concussion)” (17).

The debate is fueled by a lack of longitudinal, prospective studies of CTE and an evolving definition of CTE. While CTE has been known in the literature for nearly a century, most of the research on CTE has occurred only in the last decade. McKee et al. (18) noted that prior to 2009, there were only 48 cases of CTE in the literature, in contrast to the hundreds of cases of CTE since (19–30).

The U.S. National Institutes of Health did not recognize consensus neuropathological criteria for the post-mortem diagnosis of CTE until 2015, when an international team of neuropathologists developed the National Institute of Neurological Disorder and Stroke/National Institute of Biomedical Imaging and Bioengineering (NINDS/NIBIB) criteria, which defined the pathognomonic lesion of CTE as “an accumulation of abnormal hyperphosphorylated tau (p-tau) in neurons and astroglia distributed around small blood vessels at the depths of cortical sulci and in an irregular pattern” (31). In 2019, Falcon et al. (32) used cryo-electron microscopy to show that the misfolded p-tau filament structures of CTE are distinct from Alzheimer's and Pick's disease, providing further evidence that CTE is unique not only in terms of the composition and location of lesions but also in the shape of the misfolded p-tau. The NINDS/NIBIB criteria were updated in 2021 to refine the pathognomonic lesion to specifically require the presence of “p-tau aggregates in neurons, with or without concomitant p-tau-immunoreactive thorn-shaped astrocytes, at the depth of the sulcus around small blood vessels, in deeper cortical layers not restricted to subpial and superficial region,” which reflected the understanding that astrocytic p-tau alone was insufficient to diagnose CTE (33).

Today, CTE can only be definitively diagnosed following a neuropathological examination (31, 33). Because the National Institutes of Health recognizes consensus neuropathological criteria for CTE, but does not yet recognize clinical diagnostic criteria, this article examines the relationship between RHI and CTE neuropathology, and does not substantially explore the separate question of a causal relationship between CTE neuropathology and clinical symptoms (33). Any reference to CTE in this review refers to cases that have been confirmed by autopsy.

Developing consensus on CTE causation has important public health and medico-legal implications. If RHI causes CTE, then it becomes an environmentally caused brain disease that is preventable. If CTE is environmentally caused, then settings with exposure to RHI, which could include participation in some sports, may become regulated by governmental organizations that oversee workplace and public safety, and individuals and organizations could become financially liable for the care of those who develop CTE (34).

The largest group of individuals exposed to RHI in sports are children under the age of 18. Many scientists are concerned that children may be getting exposed to an environmentally caused neurodegenerative brain disease before the age of majority (35). Conversely, if CTE *is not* caused by RHI we could be unnecessarily changing sports with long traditions while the real causes of CTE continue undetected.

This article aims to explore the question of causation by evaluating studies exploring RHI and CTE through the Bradford Hill criteria, a widely used framework to assess causal inferences in epidemiological research.

## Understanding Causation

To determine causation in this context, it must first be understood that causation is an interpretation, not an entity (36). Outside of communicable or Mendelian genetic diseases, causation is rarely “proven” or “established,” especially for diseases involving complex environmental exposures. In these instances, causation is a continuum from highly unlikely to highly likely, and no single study can prove causation. Epidemiologists Lucas and McMichael (36) have stated causation is “merely an inference based on an observed conjunction of two variables (exposure and health status) in time and space”. Many criteria have been proposed to better answer questions of causation.

The CISG, which does not recognize a causal relationship between RHI and CTE, used the Oxford 2011 Levels of Evidence to review the evidence in the literature. The Oxford criteria were developed for clinical decision making, not for determining causation for environmental exposure (37). Thus, their conclusion that cause and effect has yet been “established” between RHI and CTE may not be based on an appropriate analysis of the evidence.

Another widely used criterion through which to examine the question of causation is the Evidence-Based Medicine Pyramid, which outlines a hierarchy of clinical evidence in considering questions of cause and effect. This framework places randomized controlled trials (RCT) as the highest levels of evidence (38), which is appropriate for evaluating the efficacy of an intervention but is not appropriate for questions of potentially environmentally mediated neurodegenerative diseases, as it would not be ethical to randomize subjects, especially a pediatric cohort, to receive RHI.

The Bradford Hill criteria were developed in 1965 by Sir Austin Bradford Hill specifically to address causation involving environmental exposures. Hill was a pioneer in the study of environmental exposures and outcomes and one of the scientists most credited with exposing the causal link between smoking and lung cancer. Hill first proposed the criteria in a lecture titled “The Environment and Disease: Association or Causation” that was later published in the *Proceedings of the Royal Society of Medicine* (39, 40). The Bradford Hill criteria, as they are commonly known, are not formal criteria or a checklist, but “viewpoints” as Hill referred to them. Evidence to support all nine viewpoints are not required to logically deduce a causal relationship, but Hill believed a statistical association should be studied through all nine viewpoints “before we cry causation” (39).

These criteria compose one of the most frequently cited frameworks for causal inference in epidemiological research and are an appropriate tool for an environmental exposure analysis.

### Strength of Association

Hill listed strength of association (SOA) first among his nine criteria, presumably because a strong association based on human epidemiologic data can dispel many other concerns about an asserted relationship between an exposure and a disease. An analysis of SOA starts from the premise that for an association to be statistically significant, the lower confidence limit (LCL) on the odds ratio (OR) or relative risk (RR) must be >1.0 (that is, very unlikely to be caused by random chance). Other things being equal, the larger the central estimate of OR or RR, the less likely that the significant association is an artifact of some *non-random* flaw such as confounding, bias, or misclassification. To the extent that the OR or RR is the “signal,” and flaws represent “noise,” the stronger the signal, the more confident we can be in presuming the variables are causally linked.

The dogma regarding SOA is that ORs/RRs that equal 2.0 or more are generally regarded as “strong.” This numerical choice is of course subjective, but the appeal of 2.0 as a benchmark is that above this ratio, it is more likely than not that any individual person with the exposure and the disease will have contracted the disease *because of the exposure* than for any other reason (in other words, if the RR is between 1 and 2, the additional risk conferred by the exposure is less than the background risk one faced without the exposure). A study exploring the relationship between NFL team logos and concussions exemplifies a reason to be wary of weak associations, as the authors found the RR of the concussion rate in the 17 “non-animal” teams was 1.34 and had a significant 95% confidence interval (41).

Cohort studies are a common method by which to identify ORs, but at this time there are no cohort studies available that have followed both symptomatic and asymptomatic individuals (to minimize selection bias) through to autopsy to confirm the presence or absence of CTE. We have identified six well-conducted case-control studies where the researchers made a reasonable attempt to identify RHI history and had more than 50 subjects to be sufficiently powered for statistical significance, brief summaries of which are provided below and in Table 1 (19, 21, 23, 42–44).

- McKee et al. (19) examined 85 brains from former athletes and others with a history of RHI from the US Department of Veterans Affairs-Boston University-Concussion Legacy Foundation (VA-BU-CLF) Brain Bank and compared them to 18 brains from age-matched control subjects with no such history. Sixty-eight of the 85 brains met diagnostic criteria for CTE, while none of the 18 control brains showed such pathology, resulting in an OR of 145. However, the OR here could be biased high, since the exposed subjects were selected for high RHI exposure.
- Bieniek et al. (23) reviewed brains from a neurodegenerative disease-focused brain bank for individuals who had had RHI. They reexamined 66 brains of individuals with prior contact-sports experience, along with 198 brains from subjects without prior contact-sports history (but 33 of these subjects had had one documented instance of traumatic brain injury; TBI). 21 of the former group, and zero of the latter, had CTE, resulting in an OR of 92.
- Adams et al. (42) examined 433 brains from two sources—the VA-BU-CLF Brain Bank that largely consists of brains donated by next-of-kin, and the Framingham Heart Study's (FHS) Brain Bank. Although their primary interest was the association of Lewy Body pathology and contact sports, their Table 1 provides ample information to estimate an OR for CTE and contact sports history. All 269 brains from the VA-BU-CLF Brain Bank had contact-sports history, and of those, 217 had CTE pathology. One brain in the FHS group had CTE, but it is unclear if they had contact sports exposure (only 12% of those in the FHS bank did so), so we can only conclude that the OR from this study is either 440 or 902.
- Bieniek et al. (43) looked at all consecutive autopsy cases during an 18-month period at the Mayo Clinic (*N* = 2,566) and gathered information about whether the subject had played one of seven different contact sports, including boxing, baseball, etc. Only the association with football history and CTE pathology had a *p*-value <0.05, and its OR was 2.62.
- Mez et al. (21) examined 266 brains from the VA-BU-CLF Brain Bank and FHS Brain Bank, 223 of which met diagnostic criteria for CTE. Although most of their analyses were aimed at calculating ORs per year of football played (e.g., their main result was an OR of 1.30 per additional year), they did offer a dichotomous OR analogous to the others in this Table. Namely, they found that subjects who played for 14.5 or more years had a CTE OR of 10.2 compared to those who played <4.5 years. This ratio is not directly comparable to the others; it could be biased high because of the very large exposures in the cases, but could also be biased low because of the non-zero exposures in the comparison group.
- Priemer et al. (44) examined 225 brains from a Department of Defense Brain Bank and found 10 of them (4.4%) with pathognomonic lesions characteristic of CTE. The OR for those with a history of exposure to blasts (e.g., from “improvised explosive devices”) compared to those without was 1.73, and not statistically significant. In stark contrast, however, the adjusted OR for CTE comparing subjects with a history of playing contact sports (including football, martial arts, wrestling, boxing, etc.) was 68.8, as there were 10 CTE cases among the 60 subjects with such history and zero cases among the 165 subjects without.

**Table 1:** Odds ratios from important case-control studies (in chronological order of publication).

**Study**	**Outcome**	**Exposure**	**OR and 95 C.I**.
McKee et al. (19)	CTE/not	hx of TBI/matched ctrls.	144.8 (8.3–2523.0)^*^
Bieniek et al. (23)	CTE/not	Contact sports/not	91.9 (12.1–701.4)^*^
Adams et al. (42)	CTE/not	hx of contact sports/not	440.1 (60.5–3203.4)^*^^,^^a^
Bieniek et al. (43)	CTE/not	hx of football/not	2.62 (1.33–5.15)^b^
Mez et al. (21)	CTE/not	Years of football (<4.5 or >14.5)	10.2 (9.8–10.7)^c^
Priemer et al. (44)	CTE/not	hx of contact sports/not	68.8 (4.0–1195.2)^*^

* *In each row with an asterisk, the raw OR was infinite because there were zero cases in the unexposed group. Following the Haldane-Anscombe correction (45), we added 0.5 to every cell in the table, yielding a finite estimate of the OR, though one that is biased low*.

a *This calculation, in order to be conservative, assumes that the one CTE case in Adams et al. (42) from the Framingham cohort did **not** have a history of contact sports. If instead we assume that that case did have contact-sports history, the OR would instead be 901.9 (55.4–14676.6)*.

b *Also arguably biased low w/respect to the NFL, because the exposed group likely had no pro athletes in it, just recreational/college*.

c *Biased low because the “unexposed” are really “low exposed”—but we see no reason to penalize this study for stratifying more finely than yes/no (they just don't have any fully unexposed, which is not a weakness)*.

After conducting our analyses, we found that all six studies reported large and significant ORs of CTE cases in subjects with substantial exposure to RHI compared to controls. There is a wide range of RHI exposure in these studies, which may account for the wide range of ORs. There is also an unknown amount of case overlap among the VA-BU-CLF Brain Bank studies.

We excluded studies (24, 25, 46–48) that did not provide the necessary raw data to estimate an odds ratio—either lacking any subjects without CTE, lacking any subjects with no exposure to RHI, or both. While it is possible to estimate an incidence of CTE in a population exposed to RHI and compare it to “historical control rates” for unexposed populations studied elsewhere, the studies referenced above were self-contained case-control investigations and hence a more reliable subset.

The clear and unremitting message from these studies is that the associations between RHI (and reliable proxies for it) and CTE cases are strong: consistently above the 2.0 benchmark, with some above ratios of 10:1 and even 100:1. Moreover, one study that included a possible confounder, Noy et al. (49) and substance abuse, showed a stronger association between the possible confounder and CTE when head trauma is present than when absent. Noy et al. were not considered in our larger analysis because RHI history was not thoroughly explored.

Importantly, relative risk is not meaningful without an understanding of absolute risk. Any “small” relative risk can correspond to a large and/or intolerable increment of absolute risk, especially when the background risk is already large. Conversely, a very large relative risk may in fact be trivial if the increment merely doubles (or multiplies by a large number) a vanishingly small absolute risk.

Two studies have provided a window into absolute risk in one highly exposed population, NFL players. In 2017, the Boston University CTE Center published that of the first 111 brains donated from former NFL players, 110 of them had CTE. Using those 110 subjects with CTE as the numerator, and the 1,142 NFL players who died during the study period (February 2008–May 2016) as the denominator, Binney and Bachynski concluded the minimum prevalence of CTE is 9.6% among NFL players (50). Alternatively, Finkel and Bieniek (51) selected for their denominator the 711 players who entered the NFL between 1963 and 2008 and died by June 2014, concluding a 15.5% prevalence was an accurate snapshot of the current prevalence of death with CTE among all NFL players who had already died of any cause.

Even if a relative risk is “small” and the absolute risk is not intolerably large, individuals have a right to be concerned about additional involuntary risks that may not be as large as their pre-existing risk from all other sources. For these reasons and others, reflexive adherence to an arbitrary criterion for how large a relative risk ought to be is not recommended.

Even a single study with a ratio of ≥2 is often ample for a regulatory agency to conclude that a particular agent (e.g., a carcinogen) is “known to be harmful to humans”—and here we have six studies with ratios far >2, backstopped by a study that controls for an important co-exposure. In the context of dozens of decisions regulatory agencies such as the Environmental Protection Agency (EPA), the Occupational Safety and Health Administration (OSHA), and the Consumer Product Safety Commission (CPSC) have made over decades, it is clear that the epidemiology of RHI and CTE is already far more definitive than many associations we have confidently deemed to be “real” for purposes of exposure reduction.

The Oxford Center for Evidence Based Medicine, which developed the Oxford Levels of Evidence used in the CISG assessment of the (lack) of evidence around CTE, states in its explanatory notes that “There will inevitably be cases where ‘lower level’ evidence – say from an observational study with a dramatic effect – will provide stronger evidence than a ‘higher level’ study – say a systematic review of few studies leading to an inconclusive result” (52). It therefore is clear that each of the above studies, showing dramatic ORs for RHI exposure in CTE, would also be considered as ‘strong evidence’ for a causal association by any reasonable interpretation of the Oxford Levels.

Further support for a robust and substantial strength-of-association comes from a different group of studies: those comparing specific or general neurodegenerative causes of death (COD) among subjects exposed to RHI vs. the general population (53–58). These studies may be capturing endpoints broader than CTE alone, but they support a causal relationship between RHI and a set of neurodegenerative diseases. Some of these studies have innovated with respect to the choice of comparison group, often in order to minimize the downward bias in risk ratio that comes from ignoring the “healthy worker effect” in study design (59). Table 2 summarizes some of the important strength-of-association results from these investigations.

**Table 2:** Other important strength of association results.

**Study**	**Outcome**	**Exposure**	**Hazard ratio**	**Notes**
Lehman et al. (53)	Neurodegenerative COD	Former NFL v. general population	3.26 (1.9–5.22)	3.26 is biased low, given that SMR for all COD combined was 0.53 (a strong “healthy worker effect”)—neurodegenerative COD were especially enriched in the NFL group
Venkataramani et al. (54)	Neurodegenerative COD	Former NFL v. former “replacement players” who played several games during the 1987 strike	4.09 (0.23–73.3)^*^	Non-significant OR. HWE minimized by using unexposed group fit enough to be on an NFL roster briefly; however, these (181) deaths are only those in each cohort who died in their 50s (about 5% of each group)
Mackay et al. (55)	Neurodegenerative COD	Former pro soccer players v. age/sex matched controls	4.1 (2.88–5.83)	
Nguyen et al. (56)	Neurodegenerative COD (underlying or contributing cause)	Former NFL players v. former Major League Baseball players	2.99 (1.64–5.45)	HWE eliminated by comparing athletes with/wo RHI. But only about 15% of this cohort had died as of study publication
Russell et al. (57)	Neurodegenerative COD	Former pro soccer players v. age/sex/SES matched controls	3.66 (2.88–4.65)	HR among defenders was >2.5 × that among goalies, suggesting a gradient with RHI
Daneshvar et al. (58)	ALS diagnosis	All NFL players who debuted between 1960 and 2019 vs. general population rates	3.59 (2.62–5.69) (incidence ratio)	3.94 (2.62–5.69) (mortality ratio)

* *Infinite raw OR (zero cases among “unexposed”); Haldane-Anscombe correction applied*.

### Consistency

Hill's consistency criterion requires that the association between RHI and CTE pathology is demonstrated across multiple studies conducted by independent groups investigating different patient populations using different approaches. CTE pathology has indeed been reported by independent investigators with varied selection criteria worldwide in post-mortem studies of individuals with exposure to RHI.

The Boston University CTE Center has reported the vast majority of CTE cases in the current literature due to the extensive collection of brains donated to the VA-BU-CLF Brain Bank, where most individuals had significant exposure to RHI. In this brain bank cohort, CTE pathology has been found in former athletes at amateur and professional levels from American football, soccer, rugby, ice hockey, lacrosse, mixed martial arts, wrestling and boxing, as well as military veterans, victims of domestic violence, an individual who suffered multiple falls and an individual who suffered from head-banging behavior (19, 22). Similarly, investigators at other North American institutions have published studies that describe the presence of CTE pathology in military veterans (60) and former athletes in American football (10, 23, 24, 61–64), amateur and professional wrestling (23, 65), and ice hockey (24). Investigators have also reported CTE pathology in Scotland (Glasgow TBI Brain Bank) (28, 48, 66), England (Queen Square Brain Bank for Neurological Disorders, University College London) (29, 67, 68), Ireland (Dublin Brain Bank) (27, 69), France (GIE Neuro-CEB Brain Bank) (30), Australia (Australian Sports Brain Bank) (25, 70, 71), Brazil (Biobank for Aging Studies at the University of São Paulo) (26), and South Korea (72). In these reports, CTE pathology was described in the brains of former soccer, rugby, Australian rules football, and boxing athletes as well as a young individual who exhibited head-banging behavior (72). This growing congruous evidence from independent investigators profiling individuals with a range of RHI exposure profiles is certainly in agreement with Hill's consistency criterion. Table 3 shows the largest CTE case series published at the Brain Banks mentioned above.

**Table 3:** CTE cases diagnosed globally.

Brain bank/group^*^	**Largest case series published**	**Publication**
UNITE/VA-BU-CLF	366^**^	Alosco et al. (73)
Queen's Square Brain Bank	32	Ling et al. (67)
Mayo Clinic Brain Bank	21	Bieniek et al. (43)
Canadian Concussion Center	17	Schwab et al. (24)
Glasgow TBI Brain Bank	12	Ameen-Ali et al. (74)
Australian Sports Brain Bank	12	Suter et al. (25)
DoD/USU Brain Tissue Repository	10	Priemer et al. (44)
University of Washington	3	Postupna et al. (75)
Biobank for Aging Studies at University of São Paulo	1	Grinberg et al. (26)
Dublin Brain Bank	1	Stewart et al. (27)
GIE Neuro-CEB Brain Bank	1	Lepreux et al. (30)
Seoul National University Hospital Brain Bank, South Korea	1	Lee et al. (72)

* *List only includes groups understood to be using NINDS/NIBIB consensus criteria for diagnosis*.

** *Boston University CTE Center website reports more than 700 diagnosed cases*.

A large proportion of the CTE literature comprises studies describing CTE pathology in a small number of individuals that have an established history of RHI and dementia symptomology. Studies that assess the presence of CTE pathology within larger brain bank cohorts further support the consistency of the association with RHI. In addition to the six case-control studies discussed in Strength of Association, a study of the Veteran Affairs ALS Biorepository Brain Bank in Boston by Walt et al. (60) found 9/21 military veterans with ALS and exposure to TBI or RHI had comorbid CTE pathology. However, some of the most robust evidence for the consistency of the association is provided by ongoing prospective or retrospective profiling of general brain bank cohorts. Ling et al. (67) screened a cohort of recent brain donations to the Queen Square Brain Bank for CTE pathology. This cohort predominantly featured cases where another neurodegenerative disease was the primary diagnosis and identified exposure to RHI in 93.8% of CTE cases (30 of 32) through post-mortem interviews with next of kin. In contrast, the brain bank study by Noy et al. (49) profiled 111 brain donations to the Health Sciences Center in Winnipeg, Manitoba, Canada, and excluded brains over 60 years of age or with any histological evidence of Alzheimer's disease. In this younger cohort without comorbid pathology, 5 cases were identified with stage I or II CTE pathology, all of which had a history of head trauma, although RHI history was not known for the subjects (49).

Together, these brain bank studies highlight that exposure to RHI from various sources is a conserved factor between individuals with CTE pathology, regardless of whether other comorbid pathologies are present. Overall, current literature from independent researchers and brain banks around the world provides strong support for Hill's consistency criterion. Future studies investigating CTE pathology in other general brain bank populations would further strengthen the evidence for the consistency of the association with RHI.

### Specificity

According to Hill, a factor is likely to be causative of disease if the disease occurs within a specific population and/or at a specific anatomical site with no reasonable explanation other than involvement of the factor in question. An absence of specificity cannot rule out causative relationships under this criterion, but when present, the greater the specificity of association between factor and outcome, the greater the probability of a causal relationship. Specificity is thus arguably one of the most persuasive of the Bradford-Hill criteria as, stated by Hill himself, “if specificity exists we may be able to draw conclusions without hesitation” (39).

Available evidence on the relationship between RHI (the factor) and CTE (the potential outcome) provides strong support for causation under this criterion. Specificity can be observed in a number of ways, but perhaps most significantly by the fact that prior exposure to RHI is the *only* known unifying factor among CTE cases reported in the literature to date (76). Neuropathological characterization of CTE began initially in former boxers in the 1970s (3), and over the ensuing three decades similar neuropathology was reported in many more ex-boxers, as well as individuals who did not have a history of boxing but were nonetheless exposed to RHI, either by occupation or underlying medical conditions. These early reports of CTE in non-boxers included those who played soccer, mentally impaired individuals prone to head banging, an individual who participated in circus “dwarf-throwing,” an individual with intractable epilepsy and frequent falls, and a victim of domestic violence (4, 77–79).

The seminal reports of CTE in two American footballers in the mid-2000s (10, 80) naturally focused investigation onto the brains of past players of this hugely popular sport, and CTE has now been reported in hundreds of ex-players of American football and 10 additional contact and collision sports (discussed in Consistency). In addition to those individuals with athletic exposure to RHI, CTE has since also been documented in multiple military personnel who were exposed to RHI either via concussion or blast injury either in combat or training exercises (81, 82).

Even more can be learned about this association by assessing the evidence for CTE neuropathology in individuals who have never been exposed to RHI. In contrast to the ever-mounting reports on CTE neuropathology in the setting of prior RHI, there are very few reports of CTE outside of it. While an absence of evidence does not equate to evidence of absence, it is remarkable that the neuropathology of CTE does not often appear in neuropathology studies, particularly in case series of individuals with neurodegenerative symptoms. For example, CTE pathology was not reported in any of the 83 brains used in Braak and Braak's staging of Alzheimer's disease pathology using Tau immunohistochemistry (83) or in any of the 198 individuals without exposure to RHI in the Mayo Clinic neurodegenerative disease Brain Bank (23).

In contrast to the prevailing literature, one recent study reported “mild” CTE in four of five men with no known history of TBI or RHI (47); however this was subsequently suggested to represent misdiagnosis of ARTAG as CTE based on the images provided in the study when reviewed by an international group of ARTAG and CTE experts (84). This conclusion is supported by the observation that although the four subjects were between the ages of 70 and 82, they had low-stage CTE, a rare finding in that age group, as CTE severity is strongly associated with age (73). ARTAG, as its name suggests, is associated with age and can mimic CTE in the location of lesions, but ARTAG does not involve neuronal tau and is not related to head impact exposure (84). This is not to say, however, that glial p-tau pathology is of no consequence in CTE, or that concomitant ARTAG is unrelated to CTE. We note that ARTAG is often found alongside CTE when glial p-tau is present in a lesion; this is remarkable, particularly when found in younger individuals in whom a diagnosis of ARTAG would be very unusual. The relationship between ARTAG and CTE clearly warrants further examination.

Another larger post-mortem study of 310 older individuals found that while ARTAG was frequently found in the general aging population (incidence of 38%), there was no evidence of CTE neuropathology in any individual (46). Postupna et al. reviewed 532 consecutive autopsy cases from the Adult Changes in Thought (ACT) study which consisted mostly of individuals with no TBI history and found only three cases of CTE (75). Two cases had a history of falls later in life and the third case had a history of military service. These studies further demonstrate the rarity of CTE diagnosis in community-based samples.

RHI is the only common denominator in reported instances of CTE to date, which indicates a specific relationship between the two. However, while CTE is closely tied to a history of RHI, this association does not imply the reverse (i.e., that all individuals who suffer from RHI will develop CTE). The specificity of the association is not one-to-one, and as Hill pointed out, such relationships are infrequent. Hill also asks us to “keep in mind that diseases may have more than one cause,” (39) and we must also consider that a factor may be causative in more than one disease.

The absence of any other plausible factor within the available evidence does not mean that CTE might not have more than one cause. If there prove to be additional causes of CTE beyond RHI exposure, they do not diminish the importance of acknowledging the relationship between RHI and CTE. For example, despite multiple non-smoking environmental, genetic, and sporadic causes of lung cancer, smoking is still considered to cause lung cancer. The presence of patients with no smoking history who develop lung cancer does not diminish the strength of relationship between smoking and lung cancer. However, the fact that a second potential cause of CTE has yet to be identified is notable, as *something* has to be causing CTE.

Taking all of the evidence together, we can conclude that specificity certainly exists for the association between RHI and CTE; RHI is the only factor common to reported CTE cases, and there is almost no evidence of CTE in those examined who have not sustained RHI. According to Hill, this should lead us to consider that the relationship between RHI and CTE is indeed a causal one.

### Temporality

A temporal relationship is a critical component to establishing a causal relationship; namely, an exposure necessarily must precede its purported outcome. RHI exposure clearly precedes CTE pathology, thus fulfilling this criterion. As outlined in the above section on Specificity, the exposure to RHI is associated with CTE pathology and, especially with the introduction of the aforementioned revised NINDS/NIBIB neuropathologic criteria requiring neuronal involvement in the perivascular deposition of tau (33, 85), this pathology occurs nearly exclusively in the presence of clearly identified RHI exposure (23, 46, 60). The RHI exposure experienced by nearly all individuals diagnosed with CTE occurred before the observed pathology, typically years if not decades earlier (19, 20, 22–24, 73, 86–90). As such, the pathology of CTE is considered a long-term consequence of RHI exposure (89, 90).

However, the pathology of CTE can only be diagnosed post-mortem, and it is unknown precisely when this pathologic process manifests. Given the wide age-range of individuals found to have CTE pathology, it is likely that CTE pathology begins significantly before it has been observed post-mortem. It should, therefore, be considered whether this pathology could manifest prior to the RHI exposure, and lead to RHI exposure in a reverse-causal manner. However, there are several factors that make this scenario exceedingly unlikely. First, as previously outlined, there is an absence of CTE pathology (using the most recent NINDS/NIBIB criteria) in individuals without exposure to RHI (23, 46, 60). If the pathology preceded exposure, one would expect to find more cases of CTE pathology in the absence of exposure, due to some individuals passing away prior to experiencing RHI. Second, although neurologic and neurodegenerative diseases are known to alter behavior, in some cases promoting both riskier behaviors as well as gait instability and falls, there is no evidence to support a neurologic phenotype for individuals who participate in contact and collision sports. Additionally, given the millions of athletes who participate in contact and collision sports annually (91), such a phenotype would be either exceedingly common, or rarely associated with CTE pathology. Finally, the pathology associated with CTE appears more severe as a function of time since injury (19–21, 73), as do biomarkers thought to be associated with this pathology (92–95), supporting a progressive pathologic process initiated by RHI exposure.

Additional evidence in support of the temporal relationship between RHI exposure and CTE comes from the clinical manifestation of CTE: traumatic encephalopathy syndrome (TES). All criteria for TES proposed to date, across multiple research groups, require a history of exposure to head injuries, either characterized as RHI, TBI, concussion, or subconcussive injuries (96–100). In addition, the symptoms thought to be most closely associated with TES also appear to manifest years following the RHI exposure (101–104). Together, these data provide overwhelming evidence supporting the temporal relationship between RHI and CTE.

### Biological Gradient

One of Hill's strongest criteria involves a dose-response association. Hill said, “If the association is one which can reveal a biological gradient, or dose-response curve, then we should look most carefully for such evidence” (39).

Martland's original ringside observations of boxers in 1928 included the recognition of a dose-response between RHI and possible CTE symptoms (1). He realized that boxers who had the greatest number of fights, who had a long duration of fighting, who were “sluggers” (taking a lot of blows), or who sparred extensively were most likely to show a clinical triad of mental confusion and recent memory impairment, emotional lability with mood swings and diminished ability to control temper, and parkinsonian signs including tremor, rigidity, and bradykinesia.

The British neurologist MacDonald Critchley was the first to publish this neurodegenerative process under the name CTE in a peer-reviewed journal in 1957 (105). He noted boxers with clinical symptoms that he believed were caused by CTE were often sluggers known for being able to “take” a punch and “absorb punishment” rather than scientific boxers. Thus, he too was describing a dose-response mechanism.

It was Corsellis who first described the neuropathology of CTE in boxers' brains that included those of two world champions and four regional or national champions. Corsellis concluded the most successful boxers had the most brain damage because they had the longest careers, fought the toughest opponents, and thus took the most blows to the head (3). Thus, this was a third independent finding of a dose exposure correlating with risk and severity of CTE.

In 1970, Friedrich Unterharnscheidt published a massive review of the historical and medical aspects of boxing in which, in addition to an exhaustive review of the medical literature published at that time, reported that “every boxer must expect permanent traumatic damage which is greater the earlier he begins to box, the more frequently he participates, and the longer his career” (106). Once again a dose-response association was recognized between RHI and CTE.

When Mez et al. (21) looked at the risk of CTE in American football players in the VA-BU-CLF and Framingham Heart Study Brain Banks, they found that years of American football played demonstrated a strong dose–response relationship with CTE neuropathology. Years of football played was used as a crude proxy for RHI, as studies using helmet sensors have shown football players receive hundreds up to a few thousand head impacts exceeding 10 g per season.

Specifically, the Boston University CTE Center researchers showed that each year of additional football play was associated with an increased OR of 1.30 (95% CI = 1.19–1.41; *p* = 3.8 × 10^−9^), effectively doubling the odds of disease every 2.6 years. The study also found that each additional year of football play was associated with an increased OR of 1.14 (95% CI = 1.07–1.22; *p* = 3.1 × 10^−4^) for having more severe CTE, effectively doubling the odds of having severe (vs. mild) CTE every additional 5.3 years of play.

Interpreting SOA here is more complicated as it stratifies exposure into single additional years played instead of a yes/no dichotomy. When exposure is parsed into multiple strata, the associations between adjacent categories can fall below 2.0, but the overall relationship between the highest and lowest exposure category can be significantly higher. In this study, players who played 14.5 years or more were 10 times more likely to have CTE than someone who played fewer than 4.5 years. In cases like this, a “weak” but consistent biological gradient bolsters a verdict of causality. Even under conditions of simulated extreme brain bank selection bias, the estimated magnitude of the relationship between duration played and CTE status remained consistent.

A major criticism of brain bank findings on CTE is the selection bias that inherently comes from this type of study design. To address this criticism, LeClair et al. evaluated the relationship between level of play in American football and CTE diagnosis after statistically adjusting for selection bias (107). From a sample of 290 participants that played at least 1 year of American football at the high school, college, or professional level, the researchers found that highest level of football play was associated with CTE diagnosis in a dose-response manner (after adjusting for age of death). In this analysis, players at the college level had 1.82 times the risk of being diagnosed with CTE than high school players, whereas those who played at the professional level had 2.13 times the risk than high school players. Following an adjustment for selection bias, these relative risks were increased in magnitude; relative to high school players, college players had a mean RR of 2.38 and professional players had a mean RR of 2.47. These findings suggest that, in this sample, selection biased the measures of association toward the null, not the alternative hypothesis as has been suggested by critics of this type of research.

To further minimize the influence of selection bias researchers have screened for CTE in community-based samples. Bieniek et al. (43) also found a dose-response relationship between exposure and observed CTE pathogenesis among football players. In this study, the authors examined tissue from 750 of 2,844 consecutive autopsy cases from the Mayo Clinic Tissue Registry for CTE pathology. They attempted to gather sports participation information through high school yearbooks and obituaries, and their sample included 450 individuals who had no record of participation in contact sports and 300 contact sport athletes. The sample included 477 males and 273 females. CTE pathology was found across a variety of sports in this sample. While college football players were significantly more likely to develop CTE than controls, high school football players, who generally play fewer years of football, were not significantly more likely, again providing evidence of a dose-response relationship.

Football and boxing are not the only sports where a dose-response relationship has been reported. Abdolmohammadi et al. presented findings at the American Academy of Neurology 74^th^ annual meeting in April 2022 that demonstrated a dose-response relationship between duration of ice hockey play and CTE risk and severity (108). Specifically, in a sample of 74 consecutive brain donors to the VA-BU-CLF and FHS Brain Banks, each additional year played corresponded to a 23% increase in odds of being diagnosed with CTE and a 15% increase in odds of increasing one stage of CTE severity (0–IV). When controlling for hockey as the primary sport of exposure (*n* = 56), similar results were found indicating that increased duration of ice hockey play may increase risk for CTE in a similar manner as was previously described in American football.

Thus, from its first description nearly 100 years ago to the present, CTE has been recognized as having a strong biological gradient, one of Hill's most salient criteria, in boxing, American football, and ice hockey.

### Plausibility

Hill's plausibility criterion requires that the association between RHI and CTE pathology can be explained in the presence of existing biological or social models. The criterion is satisfied if the relationship is consistent with the current body of knowledge regarding the etiology (RHI) and mechanism of disease (CTE) pathology. Although the state of knowledge on CTE is still incomplete, current literature supports the biological plausibility of RHI causing CTE.

The latency between RHI and the emergence of CTE has raised questions about the biological plausibility of the association, but experimental studies and computational models shed initial light on the mechanisms that may be occurring between RHI exposure and CTE pathology. While studies of non-gyrencephalic animal brains cannot produce the CTE lesion, a 2015 TBI mouse model produced p-tau immediately after a TBI, followed by later production of pathological tau deposits in the brain, while antibody-blocking of p-tau prevented tauopathy development. The same study demonstrated p-tau in human brains with CTE but not in controls (109).

One of the hallmarks of CTE pathology is the accumulation of p-tau around small blood vessels in the depths of cortical sulci (31). While investigating the biomechanics of brain injury, recent work leveraging advanced computational models have predicted patterns of brain tissue changes that correspond to characteristic CTE pathology (110–114). In 2017 Ghajari et al. (114) demonstrated that a Finite Element (FE) model of TBI predicted high levels of strain within the depths of the sulci following an American Football head collision, a fall, and a motorcycle accident. Notably, this study found that both strain and strain rate were elevated following an accident and a football collision, while only strain was elevated following a fall. This is of importance as the authors hypothesize that both strain and strain rate must pass certain thresholds for neurodegeneration to occur (115). Others have used simplified biofidelic phantoms of the human brain to model the effects of an impact and found similar results: high strain concentrated at the sulcal depth (112), demonstrating that the relationship between RHI and CTE neuropathology is plausible.

Prior animal and computations models appear to be validated by the recent discovery of CTE lesions in animals with gyrencephalic brains exposed to RHI through combative headbutting in the wild. Ackermans et al. analyzed postmortem brains of muskoxen and bighorn sheep for p-tau and other markers associated with TBI and neurodegeneration (116). They found p-tau immunoreactive structures in a distribution pattern reminiscent of early-stage human CTE with structures concentrated at the depths of the cortical sulci and occasionally around blood vessels. This study provides the first evidence that naturally occurring RHI is associated with CTE-like p-tau pathology in a gyrencephalic brain animal model.

In summary, these independent studies provide evidence of a credible mechanistic hypothesis for the location of the pathognomonic lesion, and the association between RHI and CTE alongside the paucity of CTE cases in individuals not exposed to RHI all support that RHI exposure is a plausible cause of CTE.

### Coherence

The seventh criteria, coherence, requires that the interpretation of the data not seriously conflict with what is already known about the disease or exposure. As such, here we must demonstrate that the association between RHI and CTE pathology does not conflict with what we know about the development of CTE pathology or RHI. Many of the arguments supporting the coherence of this association have been touched on in depth in previous sections of this review, including location of the pathognomonic lesion and the absence of CTE cases in individuals without exposure to RHI or with only a single TBI exposure.

Another example of coherence is found in the extreme difference between the number of known CTE cases among males and females. Bieniek et al. studied 750 brains of athletes and non-athletes, and identified 21 cases of CTE, all in males (43). Their experience is consistent with the experience of all other brain banks: while hundreds of male football players have been confirmed to have CTE by Dr. Ann McKee at the VA-BU-CLF Brain Bank alone, CTE has yet to be diagnosed in a female athlete worldwide (81).

Similar evidence in the early smoking and lung cancer data was interpreted to be a reflection of different rates of smoking among males and females. A “time trends sex differential” and “sex differential” were cited in the 1964 and 1982 United States Surgeon General reports, respectively, as evidence to support a causal relationship with several types of cancer (117).

The “sex differential” in CTE cases may reflect differences in sports RHI exposure. Sex-specific rules for sports like ice hockey and lacrosse, where body checking is illegal for females, have limited RHI exposure for females versus males (118). Historical sex differences – and discrimination – in sports participation means there are few female former contact sports participants in America over the age of 60. Females were not provided with an equal opportunity to participate in school sports until a federal civil rights law, Title IX, was enacted in 1972. CTE is primarily diagnosed in older ex-athletes with dementia, and in America, most women, even with significant RHI exposure, are potentially too young to develop a dementing brain disease that might lead to their brain being donated to a brain bank studying CTE (81). In addition, career duration for women has been limited by a lack of professional sports opportunities that endures today.

By these considerations alone, more males have athletic exposure to RHI, a greater duration of athletic exposures, and more years since RHI exposure. Additionally, the primary exposure to RHI for the majority of CTE cases has been American football. Although not technically exclusive to males, relatively few females play American football; females more commonly play touch or flag versions of the game, which are associated with less RHI exposure (119–121).

Another way to evaluate coherence is to explore alternative hypotheses. Many variables have been highlighted in the literature as potential confounders, including substance abuse, mental health disorders, genetics, and general lifestyle factors like diet and exercise (16). Although most of the candidate third variables would be expected to be disease modifying or influence the phenotype, just as they frequently do with Alzheimer's disease and other neurodegenerative processes, only one variable has been proposed as a potential alternative cause of CTE: opiate misuse. The lead author of the CISG statement has proposed that opiate misuse could completely account for CTE, writing, “The correlation between opioid abuse and hyperphosphorylated tau deposition is well described, and should be factored as a key variable in any assessment of causation [in CTE]” (122).

This argument does not hold up to scrutiny. Although tau hyperphosphorylation has been observed in the brains of individuals with a history of heroin abuse, tau deposition from opiate use is easily distinguished from the pathognomonic CTE lesion (123). While biases in selection or measurement and confounders must continue to be thoroughly explored, they do not appear to explain the RHI/CTE association at this time.

The role of genetics in CTE is beginning to be explored. A recent review on the genetics of CTE identified that several genes may play a role in modifying disease severity, including APOE, MAPT, and TMEM106B, but none have been linked to an increase risk of developing CTE. Thus far, no genes have been shown to influence the odds of developing CTE (124).

With what is known in the literature about computer modeling of brain trauma, post-mortem confirmed cases without a history of RHI exposure, sex differences, opiate use, and CTE genetics, RHI remains the only candidate risk factor for CTE causation.

### Experiment

Hill recognized that his eighth viewpoint, experiment, can be difficult to study in the context of environmental exposures. Hill wrote, “Occasionally it is possible to appeal to experimental, or semi-experimental, evidence. For example, because of an observed association some preventive action is taken. Does it in fact prevent?” (39). If CTE is caused by RHI, sports can offer a laboratory for CTE prevention efforts. However, CTE has only been widely recognized outside of boxing for fewer than 20 years, which does not allow for meaningful studies of CTE prevention efforts. It is also too soon to measure the potential impact of recent efforts made to reduce RHI, and it will still be difficult to measure 50 years from now, as the same changes are often not uniformly made across age groups and leagues. For example, while the NFL Players Association negotiated to limit RHI by limiting contact in practice in the NFL, practices are not regulated to the same extent, if at all, at the youth, high school, and collegiate levels, which make up most of an NFL player's lifetime exposure to RHI (125).

Even in scenarios where we may have had enough time to measure the impact of an intervention, the inability to measure or control all variables of interest can make it difficult to draw conclusions. For example, a 1983 rule change to shorten boxing matches from 15 to 12 rounds has been credited with making boxing safer. However, an analysis of mortality after the 1983 reform concluded mortality reductions were more influenced by overall shorter fights, fewer fights per fighter, and shorter careers; thus their impact on CTE prevalence is unknown (126). In addition, a confounder for any study of RHI in boxing is RHI from sparring, which can make up the majority of RHI exposure in the career of a boxer but is generally not measured. Because CTE can only be diagnosed post-mortem, the latency will make drawing any conclusions nearly impossible.

Pre-clinical evidence from *in vivo* studies contribute to Hill's assertion that evidence drawn from experimental manipulation interposes support for causal inference in the links between repetitive physical trauma and chronic neurodegeneration (40). Studying animal models enables broader experimental frameworks to be used to address clinically relevant questions and importantly enhances the ability to effects in controlled environments (i.e. in the absence of extrinsic factors).

It can be argued that studies of animal models enable research questions to be answered in a more direct manner compared to human studies. However, it is important to note that the elucidation of the data generated from these studies contributes to the criterion by which experimental and interpretive validity is established (127). As asserted by Goldstein et al. (127), validation is not presented on that animal model *per se*, but rather on the quality and interpretation of experimental data arising from a model and on the experimental agreement of the model with clinical features and pathology of the human disorder that is being modeled (127–129).

Clinical studies in mild brain injury have traditionally been criticized for their observational and retrospective design which potentially affects outcomes and interpretation of research findings due to confounding factors such as the individuals' lifestyle and choices they make. Animal models are essential for translational research in our understanding of CTE, but they are not without their own unique limitations (127). For example, rodent brains lack gyri or sulci, so they cannot recapitulate the human CTE lesion.

Methodological approaches in clinical and preclinical study designs have evolved as our questions regarding links between repeated neurotrauma and neurodegenerative diseases have similarly progressed. Pre-clinical study designs have now started using repeated mild TBI (rTBI) and multiple mild TBI (mmTBI) models to understand the effects of subtle but repetitive impacts on long-term neurological disease. For the purposes of this discussion, rTBI refers to two separate insult events, whereas mmTBI involves three or more insult events.

Studies investigating the time between repeat impacts demonstrated greater effects on tissue damage when the repeated impact was delivered within a shorter time period, demonstrating vulnerability of the brain following a mTBI (130–133).

More recent studies using a model of repetitive insults (mmTBI) have demonstrated pathophysiology (134), and pathology in rats experiencing multiple impacts elevated p-tau immune reactivity (135) and neurotoxic tau (136). Other groups, including Mouzon et al. (137), have not detected abnormal p-tau after mmTBI.

Pre-clinical models provide an effective pathway for translation of basic science discovery progressing clinical knowledge that benefit human participants (127, 138, 139). Studies have shown neurobehavioral, pathophysiological and pathological alterations after a single traumatic insult; but despite some variability in results in repeat insult models reflecting methodological differences, the overall data demonstrates far worse longitudinal outcomes following rTBI and mmTBI. Through Hill's criterion of experiment, the findings from pre-clinical RHI studies replicating elements of observed human pathophysiological (140, 141), imaging (142, 143), and epidemiological studies (21) suggests a causal relationship.

### Analogy

The ninth and final viewpoint proposed by Hill is that of analogy. It is articulated that ‘in some circumstances it would be fair to judge by analogy’ whereby scientists can use prior knowledge and patterns to infer similar causal associations (39). While some have argued that analogy is not necessary in arguing causation, it does engender a sense of credibility through resembling other accepted scientific truths (40).

The evidence supporting TBI as a causal risk factor for dementia is analogous to the relationship between RHI and CTE (144). The 2020 Lancet Commission review on Dementia Prevention, Intervention and Care highlighted that the prevention of TBI in mid-life is a key strategy for the prevention of dementia (145). While the differences between TBI and RHI models are outlined in the plausibility and experiment sections, pre-clinical studies suggest repeated and multiple RHIs result in more pathologies than a single impact.

The discovery of the link between smoking and environmentally caused lung cancer is analogous to the accruing body of evidence linking RHI to CTE. Both links were controversial, specifically because the extreme latency between exposure and symptomatic disease. In fact, the Bradford Hill criteria was partly built on Hill's experience demonstrating a causal relationship between smoking and lung cancer. The early smoking and lung cancer data included a significant strength of association, a biological gradient, coherence, plausibility, and temporality, but it took time to build evidence through consistency, specificity, and experiment. It is worth noting that Sir Richard Doll, a colleague of Hill's, concluded a causal relationship between smoking and lung cancer even before he understood which specific carcinogens in cigarette smoke were causing the cancer, “for the human evidence was so compelling” (146).

More important to consider, however, is the level of evidence that has been obtained to achieve what has been described as a “well-established” causal link between smoking and the risk of lung cancer (147). While the link between smoking and lung cancer is widely accepted, key questions remain unanswered or incompletely answered, including what precisely constitutes a smoked cigarette (the dose), why some smokers develop cancer and others do not, how many cigarettes are too many, or which specific cigarette or carcinogen sparked the lung cancer.

The fact that these questions also remain for RHI and subconcussive impacts is often raised as a reason that conclusions on RHI/CTE causation cannot be drawn. The CISG wrote:

“The challenge researchers face at this time is that (i) there is no established definition of a subconcussive impact or a subconcussive injury, (ii) a [sic] impact may or may not cause an injury, and it is difficult to determine if an injury has occurred, and (iii) the biomechanical features and thresholds for quantifying a [sic] impact and identifying an injury have not been agreed upon. Therefore, the hypothesis that subconcus-sive impacts cause long-term neurological injury requires more research before conclusions can be drawn” (16).

One could imagine this passage rewritten on smoking and lung cancer today:

“The challenge researchers face at this time is that (i) there is no established definition of a smoked cigarette (ii) a single cigarette may or may not cause pre-cancerous changes, and it is difficult to determine if pre-cancerous changes have occurred, and (iii) the biomechanical features and thresholds (e.g., depth of inhalation, percent of cigarette smoked, puffs per cigarette, total duration per cigarette, specific ingredients, presence of filter) for quantifying a smoked cigarette have not been agreed upon. Therefore, the hypothesis that cigarettes cause lung cancer long-term requires more research before conclusions can be drawn” (16).

These knowledge gaps have not limited the ability to assert a causal link between smoking and lung cancer, and similarly should not limit the ability to determine the likelihood of a causal link between RHI and CTE.

## Discussion

The Bradford Hill criteria were created to help scientists transition from an observed association between two variables to a verdict of likely causation. These criteria were developed in part from Hill's experience investigating a causal relationship between cigarette smoking and lung cancer, and are among the most frequently cited metrics for evaluating causal inference in epidemiology. Hill said, “None of my nine viewpoints can bring indisputable evidence for or against the cause and-effect hypothesis and none can be required as a sine qua non. What they can do, with greater or less strength, is to help us to make up our minds on the fundamental question—is there any other way of explaining the set of facts before us, is there any other answer equally, or more, likely than cause and effect?” (39).

On the fundamental question of the relationship between RHI and CTE, an exploration of the literature through each of Hill's nine viewpoints suggests an extremely high likelihood of a causal relationship. That conclusion is bolstered by the complete absence of evidence for plausible alternative hypotheses. Besides RHI, there does not appear to be any other common variable or possible mechanism that would explain why so many contact sport athletes, in diverse sports, and in multiple countries, are being diagnosed with CTE, while individuals without RHI exposure are not.

The extraordinary statistical relationship between RHI and CTE, both in strength of association as well as dose-responsiveness, supports causality. Novel data analyses among the four case-control studies that included unexposed controls revealed that the three studies with the largest share of professional and college athletes found a striking strength of association, exceeding 90:1 in all three. In the fourth study, with the least exposed population, the OR among American football players still exceeded 2.

In addition, the largest neuropathologic studies ever published of American football and ice hockey players have revealed an increased OR for developing CTE exceeding 1.2 for *each* additional year of play, on par with studies of years of smoking and lung cancer (148).

Although the dogma that an OR of 2 implies a “strong” association may be useful in certain instances (e.g., for decisions on compensation in a court case), it is not necessarily appropriate for public health broadly. For example, history is replete with smart decisions to control hazards long before they are “established.” Frances Kelsey single-handedly kept thalidomide off the US market because she responded to three isolated case reports of phocomelia in Australia (149). Tens of thousands of Americans born circa 1960 would be severely disabled today had she and FDA waited for the relationship to be pronounced “causal” based on epidemiology with RR ≥2 and other Bradford Hill boxes checked off. OSHA kept male workers from being sterilized by DBCP (dibromochloropropane) circa 1977 based on a report of eight workers with reproductive damage in one factory (150). Could this have been an artifact? Quite possibly so. But the chemical was strictly regulated, at reasonable if not trivial cost, and no more case clusters like that have occurred once the fumigant industry changed chemicals.

This principle is, moreover, why OSHA has required since 1983 (Hazard Communication Standard) that chemical manufacturers disclose all worrisome findings in the medical literature, including RCTs, positive epidemiology (even if the RR <2), and animal bioassays, but also case reports (151).

There are obvious ways to observe or reliably infer causality without the delay and ethical problems of mounting an RCT. Relationships can be seen with only a small window of insight: X-rays cause cancer, and lead aprons block X-rays, with both statements verified by experiment. We did not conduct an RCT of cancer rates among patients given or withheld lead aprons during all X-rays before simply asserting that a known way to reduce a known harmful exposure must be salutary. Due in part to this history, society now takes precautionary action against a wide variety of exposures that add (far) less than the original risk to the background (that is, far less than a relative risk of two among the exposed). The Environmental Protection Agency (EPA) regulates incremental risks as low as 10^−6^, and OSHA at least as low as 10^−3^ (51). The excess absolute risks of CTE in the presence of RHI reported here exceed these thresholds by many orders of magnitude.

There are many valid reasons why some scientists remain skeptical of concluding a causal relationship between RHI and CTE. Brain bank studies can be subject to selection biases, there remains an absence of prospective longitudinal studies, the clinical phenotype is not fully defined, and the definition of CTE has changed multiple times over the last 100 years. However, these are not *prima facie* reasons to reject a conclusion of causation.

Brain bank data are always subject to selection biases, beginning with, but not limited to the fact that in most cases, the families of donors must be willing to participate. However, numerous independent research groups, using data from multiple independent brain banks with varying selection criteria, have consistently diagnosed CTE nearly exclusively in individuals with documented exposure to RHI. In addition, a recent analysis of bias in the largest CTE brain bank reported that bias may be causing an *underestimation* of risk (107).

While the clinical phenotype is not fully defined, CTE pathology is significantly and strongly associated with dementia. In the largest study of CTE cases to date (*n* = 366), Alosco et al. investigated the utility of the four-stage McKee CTE staging scheme and found that, controlling for age of death and comorbid pathologies, for every one level increase in the stage of CTE, there was a 1.64× increased odds for antemortem dementia at time of death (95% CI 1.19–2.27, *p* = 0.003) (73).

While some longitudinal studies have begun, longitudinal studies alone will not answer the question of causation. Barring an unlikely and ethically problematic study separating groups of identical twins at birth, exposing one group to RHI (ideally beginning in childhood), and protecting the other from all head impacts throughout their life, while following both groups for decades of life through death and controlling for all known and unknown variables, longitudinal studies cannot *by themselves* answer the question of causation.

The changing definition of CTE over time is inevitable in science as technology advances and discoveries are made. The current definition of CTE was made possible only by the discovery of tau protein in 1975, and the current definition of CTE has been used in the diagnosis of the overwhelming majority of CTE cases (152).

It is important to consider why the question of causation, which appeared settled throughout the 20^th^ century, is now receiving so much attention, especially given that even stronger data linking RHI to CTE has been published in the past decade. Many papers highlight the extent to which this link had been considered scientifically settled; for example, a review article from 1994 stated:

“After years of being slammed repeatedly in the head by the gloved fists of an opponent, many seasoned boxers begin to show the effects of the brutal punishment by displaying slurred speech, leg dragging, hand tremors, and mental confusion. Boxing fans long ago referred to these symptoms as the “punch drunk syndrome.” The proposition that the punch drunk syndrome is directly linked to the cumulative punishment boxers suffer is widely accepted” (153).

It is worth exploring the factors that led to this renewed debate, given the absence of recent alternative evidence or plausible explanations for CTE pathology. Undoubtedly, a determination of causation between RHI and CTE has significant medico-legal consequences for professional and amateur sports, both in terms of liability and long-term viability. Lawsuits have been filed by former players or their estates against the NFL, NHL, NCAA, World Rugby, the International Olympic Committee (IOC) and many others, and most are still being litigated (154–157). Among this group, only the NFL has reversed their stance on causation, and notably only after settling a class-action lawsuit with former players for an uncapped amount that will exceed $1 billion (158–160).

CISG, the most prominent organization that argues against a causal link between RHI and CTE, developed their statement at a meeting sponsored by the IOC, Fédération Internationale de Football Association (FIFA), International Federation for Equestrian Sports (FEI), World Rugby and the International Ice Hockey Federation. The CISG leadership is determined by those organizations (161). Of the 36 authors of the statement, 32 had ties to professional sports leagues (162). The former lead author and chair, who resigned in 2022 following plagiarism allegations, has advised the IOC, FIFA, and the Australian Football League, has received funding from the NFL, and has served as an expert witness for the NHL.

Beyond direct legal ramifications, sports organizations may be concerned that if their sport is shown to cause cases of CTE, viewership patterns may change, and parents may consider steering their children toward sports that do not result in RHI.

While current science overwhelmingly suggests RHI causes CTE, it does not mean that the debate is over. As Hill said, “All scientific work is incomplete - whether it be observational or experimental. All scientific work is liable to be upset or modified by advancing knowledge” (39).

The fact that new science could change our perspective on causality “does not confer upon us a freedom to ignore the knowledge we already have, or to postpone the action that it appears to demand at a given time,” according to Hill (39). Accepting the causal RHI/CTE link opens the door to actions that could dramatically reduce the burden of CTE moving forward, while we await scientific advances.

For those already exposed to RHI, the benefits to individuals with or at risk of developing CTE could be enormous. If causality is considered “established,” it is not hard to imagine global governments and research funding organizations focused on public health investing in diagnostics, treatments, and clinical trials. Knowing their research is less vulnerable to corporate or political influence, more scientists would be able to dedicate their careers to unlocking the remaining mysteries of the disease, make a greater commitment to clinical care (thereby supporting both patients and their caregivers), and conduct more research into the impact of CTE on spouses, children, and families.

Hill urged, “In asking for very strong evidence I would, however, repeat emphatically that this does not imply crossing every ‘t’, and swords with every critic, before we act” (39). Any actions are risky, including decisions to do nothing. When thinking of causation as a continuum from highly unlikely to highly likely, we must consider the expected net harm of doing *something* vs. doing *nothing*. If we do something, and we learn later that the hazard was not really a hazard, there may be harm done. If we do nothing, and the hazard was in fact a hazard, there will be harm done of a very different sort (163).

While professional athletes in sports like American or Australian football, soccer, rugby, and ice hockey are unlikely to suddenly quit their sports if causation is “reestablished,” they would gain informed consent about the risks associated with their participation. They would also gain insight to RHI as a risk factor for long-term brain health, which could inform future choices about RHI exposure and lifestyle, as well destigmatize seeking care for mental health symptoms. One could imagine this globally influential group of elite athletes mobilizing to accelerate CTE research and prevention efforts, to not only benefit themselves, but all impacted by CTE.

Perhaps most consequential would be the positive health impact for children. As it stands today, tens of millions of children as young as 5 years old are exposed to RHI in sports because they are playing by rules that were originally designed for adults. Armed with confidence in the causal connection between RHI and CTE, parents and youth coaches may reject exposing their children to a preventable degenerative brain disease simply because the current rules (tackling, heading) make RHI inevitable, especially when non-RHI versions of those contact sports exist, as well as alternative sports without RHI. Considering that both CTE onset and severity have been associated with a dose-response, strict reforms that lower the dose could effectively prevent new cases of the disease.

Through the lens of the Bradford Hill criteria, the scientific evidence suggests we need to immediately align public health policies with a presumption of a causal link between RHI and CTE.

## Conclusion

The evidence on the link between RHI and CTE is imperfect, and like all similar research, it will remain imperfect in perpetuity. After reviewing the medical literature on RHI and CTE through the Bradford Hill criteria, we have the highest confidence in the conclusion that RHI *causes* CTE. We encourage the medical, scientific and public health communities to now act under the premise of a causal relationship and take immediate action to prevent CTE, minimize risk, and develop therapeutics to slow or stop disease progression.

To accomplish this, we must make greater investments in research to better understand the mechanism of CTE and develop biomarkers to diagnose CTE *in vivo* and measure the effect of interventions. We need to accelerate research to advance our limited understanding of the role of genetic and non-genetic risk factors and risk modifiers in CTE outcomes, anatomic location, symptomatology, progression, and severity.

Additional research will inform prevention and therapeutic strategies for the hundreds of millions of individuals worldwide already exposed to RHI and at risk of developing CTE. However, while we call for more research, we also believe that the strength of the current evidence compels us to move past a scientific discussion focused solely on filling gaps in the evidence to focus on immediately implementing aggressive CTE mitigation programs, especially for children.

We support measures to minimize and eliminate RHI as the best action for preventing CTE. We encourage awareness efforts so parents, athletes, and policymakers can better understand the risks associated with RHI, change how games are played to reduce or eliminate RHI – especially for children – and make more informed decisions regarding participation in contact sports.

Finally, we encourage the medical, scientific, and public health community to reflect on the risks of RHI to children, a vulnerable population that cannot provide informed consent to participate in activities that may cause a preventable neurodegenerative disease. We can, and should, do what is possible to prevent children from developing CTE before they can possibly understand how CTE might impact their future.

## Author Contributions

CN, SB, and RC contributed to the conception and design of the review. AF performed statistical analysis. CN and SB wrote the first draft of the manuscript. All authors wrote sections of the manuscript and contributed to manuscript revision, read, and approved the submitted version.

## Conflict of Interest

CN is a volunteer member of the Mackey-White Committee of the National Football League Players Association for which he receives travel support and an advisor to Oxeia Biopharmaceuticals, LLC, Aurora CTS, and PreCon Health. DD provides ad-hoc expert testimony in medico-legal matters pertaining to traumatic brain injury. EH-Y is a scientific advisor for Vernx Pty Ltd. No funding was received from Vernx. AP currently receives partial research salary funding from Erasmus+ strategic partnerships program (2019-1-IE01-KA202-051555) and previously received partial research funding from the Sports Health Check Charity (Australia), Australian Football League, Impact Technologies Inc., and Samsung Corporation, and is remunerated for expert advice to medico-legal practices. RC is a paid consultant to the National Football League Head, Neck and Spine Committee, a vice president and chair of the scientific advisory committee of the National Operating Committee on Standards for Athletic Equipment, and a consultant to the Concussion Legacy Foundation; he also receives royalties from Houghton Mifflin Harcourt and compensation for expert legacy opinion to the National Collegiate Athletic Association and National Hockey League and is a volunteer member of the National Football League Players Association for which he receives travel support. The remaining authors declare that the research was conducted in the absence of any commercial or financial relationships that could be construed as a potential conflict of interest. The handling editor RA declared a shared affiliation with the author LG at the time of review.

## Publisher's Note

All claims expressed in this article are solely those of the authors and do not necessarily represent those of their affiliated organizations, or those of the publisher, the editors and the reviewers. Any product that may be evaluated in this article, or claim that may be made by its manufacturer, is not guaranteed or endorsed by the publisher.

## References

[1] Martland HS. Punch drunk. J Am Med Assoc. (1928) 91:1103–7. 10.1001/jama.1928.0270015002900925996397

[2] Montenigro PH Corp DT Stein TD Cantu RC Stern RA. Chronic traumatic encephalopathy: historical origins and current perspective. Annu Rev Clin Psychol. (2015) 11:309–30. 10.1146/annurev-clinpsy-032814-112814 25581233

[3] Corsellis JAN Bruton CJ Freeman-Browne D. The aftermath of boxing. Psychol Med. (1973) 3:270–303. 10.1017/S0033291700049588 4729191

[4] Roberts GW Whitwell HL Acland PR Bruton CJ. Dementia in a punch-drunk wife. Lancet. (1990) 335:918–9. 10.1016/0140-6736(90)90520-F 1970008

[5] Hof PR Bouras C Buée L Delacourte A Perl DP Morrison JH. Differential distribution of neurofibrillary tangles in the cerebral cortex of dementia pugilistica and Alzheimer's disease cases. Acta Neuropathol. (1992) 85:23–30. 10.1007/BF00304630 1285493

[6] Geddes JF Vowles GH Robinson SF Sutcliffe JC. Neurofibrillary tangles, but not Alzheimer-type pathology, in a young boxer. Neuropathol Appl Neurobiol. (1996) 22:12–6. 10.1046/j.1365-2990.1996.2598025.x 8866777

[7] Steinbeck J. In Dubious Battle. New York, NY: Random House (1936).

[8] Nelson R. Requiem for a Heavyweight. Los Angeles, CA: Playhouse 90 (1956).

[9] Kazan E. On the Waterfront. Los Angeles, CA: Columbia Pictures Corporation (1954).

[10] Omalu BI DeKosky ST Minster RL Kamboh MI Hamilton RL Wecht CH. Chronic traumatic encephalopathy in a National Football League player. Neurosurgery. (2005) 57:128–33. 10.1227/01.NEU.0000163407.92769.ED 15987548

[11] Burnsed B. A Gray Matter. Los Angeles, CA: NCAA Champion Magazine (2015) Spring.

[12] NCAA chief medical officer reflects on progress of concussion research. NCAA.org. Available online at: https://www.ncaa.org/sports/2019/7/11/ncaa-chief-medical-officer-reflects-on-progress-of-concussion-research.aspx (accessed May 6, 2022).

[13] Ezell L. Timeline: The NFL's Concussion Crisis – League of Denial: The NFL's Concussion Crisis. Chicago, IL: FRONTLINE (2013).

[14] Bettman G. Letter to U.S. Senator Richard Blumenthal. From: Bettman Responds to C.T.E. Concerns. The New York Times. (2016). Available online at: https://www.nytimes.com/interactive/2016/07/26/sports/hockey/27hockey-doc.html (accessed May 6, 2022).

[15] McCrory P Meeuwisse WH Aubry M Cantu B Dvorák J Echemendia RJ. Consensus statement on concussion in sport: the 4th International Conference on Concussion in Sport held in Zurich, November 2012. Br J Sports Med. (2013) 47:250–8. 10.1136/bjsports-2013-092313 23479479

[16] Manley G Gardner AJ Schneider KJ Guskiewicz KM Bailes J Cantu RC. A systematic review of potential long-term effects of sport-related concussion. Br J Sports Med. (2017) 51:969–77. 10.1136/bjsports-2017-097791 28455362 PMC5466926

[17] U.S. Centers for Disease Control and Prevention. Answering Questions About Chronic Traumatic Encephalopathy (CTE): Information for Healthcare Providers. (2019). Available online at: https://www.cdc.gov/traumaticbraininjury/pdf/CDC-CTE-ProvidersFactSheet-508.pdf (accessed April 28, 2022).

[18] McKee AC Cantu RC Nowinski CJ Hedley-Whyte ET Gavett BE Budson AE. Chronic traumatic encephalopathy in athletes: progressive tauopathy after repetitive head injury. J Neuropathol Exp Neurol. (2009) 68:709–35. 10.1097/NEN.0b013e3181a9d503 19535999 PMC2945234

[19] McKee AC Stein TD Nowinski CJ Stern RA Daneshvar DH Alvarez VE. The spectrum of disease in chronic traumatic encephalopathy. Brain. (2013) 136:43–64. 10.1093/brain/aws307 23208308 PMC3624697

[20] Mez J Daneshvar DH Kiernan PT Abdolmohammadi B Alvarez VE Huber BR. Clinicopathological evaluation of chronic traumatic encephalopathy in players of American football. JAMA. (2017) 318:360–70. 10.1001/jama.2017.16687 28742910 PMC5807097

[21] Mez J Daneshvar DH Abdolmohammadi B Chua AS Alosco ML Kiernan PT. Duration of American football play and chronic traumatic encephalopathy. Ann Neurol. (2020) 87:116–31. 10.1002/ana.25611 31589352 PMC6973077

[22] Mez J Alosco ML Daneshvar DH Saltiel N Baucom Z Abdolmohammadi B. Validity of the 2014 traumatic encephalopathy syndrome criteria for CTE pathology. Alzheimers Dement. (2021) 17:1709–24. 10.1002/alz.12338 33826224 PMC8596795

[23] Bieniek KF Ross OA Cormier KA Walton RL Soto-Ortolaza A Johnston AE. Chronic traumatic encephalopathy pathology in a neurodegenerative disorders brain bank. Acta Neuropathol. (2015) 130:877–89. 10.1007/s00401-015-1502-4 26518018 PMC4655127

[24] Schwab N Wennberg R Grenier K Tartaglia C Tator C Hazrati LN. Association of position played and career duration and chronic traumatic encephalopathy at autopsy in elite football and hockey players. Neurology. (2021) 96:e1835–43. 10.1212/WNL.0000000000011668 33627496 PMC8105967

[25] Suter CM Affleck A Lee M Pearce AJ Iles L Buckland ME. Chronic traumatic encephalopathy in Australia: the first three years of the Australian Sports Brain Bank. Med J Aust. (2022) 216:530–1. 10.5694/mja2.51420 35144312 PMC9305557

[26] Grinberg LT Anghinah R Nascimento CF Amaro E Leite RP Da Graca Martin M. Chronic traumatic encephalopathy presenting as Alzheimer's disease in a retired soccer player. J Alzheimers Dis. (2016) 54:169–74. 10.3233/JAD-160312 27472879 PMC5293758

[27] Stewart W McNamara PH Lawlor B Hutchinson S Farrell M. Chronic traumatic encephalopathy: a potential late and under recognized consequence of rugby union? QJM. (2016) 109:169–74. 10.1093/qjmed/hcv070 25998165

[28] Arena JD Smith DH Lee EB Gibbons GS Irwin DJ Robinson JL. Tau immunophenotypes in chronic traumatic encephalopathy recapitulate those of ageing and Alzheimer's disease. Brain. (2020) 143:1572–87. 10.1093/brain/awaa071 32390044 PMC7241956

[29] Ling H Morris HR Neal JW Lees AJ Hardy J Holton JL. Mixed pathologies including chronic traumatic encephalopathy account for dementia in retired association football (soccer) players. Acta Neuropathol. (2017) 133:337–52. 10.1007/s00401-017-1680-3 28205009 PMC5325836

[30] Lepreux S Auriacombe S Vital C Dubois B Vital A. Dementia pugilistica: a severe tribute to a career. Clin Neuropathol. (2015) 34:193–8. 10.5414/NP300838 25828776

[31] McKee AC Cairns NJ Dickson DW Folkerth RD Dirk Keene C Litvan I. The first NINDS/NIBIB consensus meeting to define neuropathological criteria for the diagnosis of chronic traumatic encephalopathy. Acta Neuropathol. (2016) 131:75–86. 10.1007/s00401-015-1515-z 26667418 PMC4698281

[32] Falcon B Zivanov J Zhang W Murzin AG Garringer HJ Vidal R. Novel tau filament fold in chronic traumatic encephalopathy encloses hydrophobic molecules. Nature. (2019) 568:420–3. 10.1038/s41586-019-1026-5 30894745 PMC6472968

[33] Bieniek KF Cairns NJ Crary JF Dickson DW Folkerth RD Keene CD. The Second NINDS/NIBIB consensus meeting to define neuropathological criteria for the diagnosis of chronic traumatic encephalopathy. J Neuropathol Exp Neurol. (2021) 80:210–9. 33611507 10.1093/jnen/nlab001 PMC7899277

[34] Finkel AM Deubert CR Lobel O Cohen IG Lynch HF. The NFL as a workplace: the prospect of applying occupational helath and safety law to protect NFL workers. Ariz Law Rev. (2018) 60:291–368.

[35] Bachynski KE Goldberg DS. Youth sports & public health: framing risks of mild traumatic brain injury in American football and ice hockey. J Law Med Ethics. (2014) 42:323–33. 10.1111/jlme.12149 25264090

[36] Lucas RM McMichael AJ. Association or causation: evaluating links between “environment and disease”. Bull World Health Organ. (2005) 83:792–5. 16283057 PMC2626424

[37] Parkhurst JO Abeysinghe S. What constitutes “good” evidence for public health and social policy-making? From hierarchies to appropriateness. Soc Epistemol. (2016) 30:665–79. 10.1080/02691728.2016.1172365

[38] Murad MH Asi N Alsawas M Alahdab F. New evidence pyramid. Evid Based Med. (2016) 21:125–7. 10.1136/ebmed-2016-110401 27339128 PMC4975798

[39] Hill AB. Inaugural Meeting. Proc R Soc Med. (1965) 58:289.19994393

[40] Fedak KM Bernal A Capshaw ZA Gross S. Applying the Bradford Hill criteria in the 21st century: how data integration has changed causal inference in molecular epidemiology. Emerg Themes Epidemiol. (2015) 12:14. 10.1186/s12982-015-0037-4 26425136 PMC4589117

[41] Smoliga JM Zavorsky GS. Team logo predicts concussion risk: lessons in protecting a vulnerable sports community from misconceived, but highly publicized epidemiologic research. Epidemiology. (2017) 28:753–7. 10.1097/EDE.0000000000000694 28570384

[42] Adams JW Alvarez VE Mez J Huber BR Tripodis Y Xia W. Lewy body pathology and chronic traumatic encephalopathy associated with contact sports. J Neuropathol Exp Neurol. (2018) 77:757–68. 10.1093/jnen/nly065 30053297 PMC6097837

[43] Bieniek KF Blessing MM Heckman MG Diehl NN Serie AM Paolini MA. Association between contact sports participation and chronic traumatic encephalopathy: a retrospective cohort study. Brain Pathol. (2020) 30:63–74. 10.1111/bpa.12757 31199537 PMC6916416

[44] Priemer DS Iacono D Rhodes CH Olsen CH Perl DP. Chronic traumatic encephalopathy in the brains of military personnel. N Engl J Med. (2022) 386:2169–77. 10.1056/NEJMoa2203199 35675177

[45] Ruxton GD Neuhäuser M. Review of alternative approaches to calculation of a confidence interval for the odds ratio of a 2 × 2 contingency table. Methods Ecol. Evol. (2013) 4:9–13. 10.1111/j.2041-210x.2012.00250.x

[46] Forrest SL Kril JJ Wagner S Hönigschnabl S Reiner A Fischer P. Chronic traumatic encephalopathy (CTE) is absent from a European community-based aging cohort while cortical aging-related tau astrogliopathy (ARTAG) is highly prevalent. J Neuropathol Exp Neurol. (2019) 78:398–405. 10.1093/jnen/nlz017 30939193

[47] Iverson GL Luoto TM Karhunen PJ Castellani RJ. Mild chronic traumatic encephalopathy neuropathology in people with no known participation in contact sports or history of repetitive neurotrauma. J Neuropathol Exp Neurol. (2019) 78:615–25. 10.1093/jnen/nlz045 31169877 PMC6581557

[48] Lee EB Kinch K Johnson VE Trojanowski JQ Smith DH Stewart W. Chronic traumatic encephalopathy is a common co-morbidity, but less frequent primary dementia in former soccer and rugby players. Acta Neuropathol. (2019) 138:389–99. 10.1007/s00401-019-02030-y 31152201 PMC6689293

[49] Noy S Krawitz S Del Bigio MR. Chronic traumatic encephalopathy-like abnormalities in a routine neuropathology service. J Neuropathol Exp Neurol. (2016) 75:1145–54. 10.1093/jnen/nlw092 27815395

[50] Binney ZO Bachynski KE. Estimating the prevalence at death of CTE neuropathology among professional football players. Neurology. (2019) 92:43–5. 10.1212/WNL.0000000000006699 30487144

[51] Finkel AM Bieniek KF A. quantitative risk assessment for chronic traumatic encephalopathy (CTE) in football: How public health science evaluates evidence. Hum Ecol Risk Assess. (2019) 25:564–89. 10.1080/10807039.2018.1456899

[52] Howick J Chalmers I Glasziou P Greenhalgh T Heneghan C Liberati A. The 2011 Oxford CEBM Levels of Evidence (Introductory Document). Oxford Centre for Evidence-Based Medicine. Available online at: https://www.cebm.ox.ac.uk/resources/levels-of-evidence/ocebm-levels-of-evidence (accessed May 6, 2022).

[53] Lehman EJ Hein MJ Baron SL Gersic CM. Neurodegenerative causes of death among retired national football league players. Neurology. (2012) 79:1970–4. 10.1212/WNL.0b013e31826daf50 22955124 PMC4098841

[54] Venkataramani AS Gandhavadi M Jena AB. Association between playing American football in the national football league and long-term mortality. JAMA. (2018) 319:800–6. 10.1001/jama.2018.0140 29392304 PMC5838566

[55] Mackay DF Russell ER Stewart K MacLean JA Pell JP Stewart W. Neurodegenerative disease mortality among former professional soccer players. N Engl J Med. (2019) 381:1801–8. 10.1056/NEJMoa1908483 31633894 PMC8747032

[56] Nguyen VT Zafonte RD Chen JT Kponee-Shovein KZ Paganoni S Pascual-Leone A. Mortality among professional American-style football players and professional American baseball players. JAMA Netw Open. (2019) 2:e194223. 10.1001/jamanetworkopen.2019.4223 31125098 PMC6632140

[57] Russell ER MacKay DF Stewart K MacLean JA Pell JP Stewart W. Association of field position and career length with risk of neurodegenerative disease in male former professional soccer players. JAMA Neurol. (2021) 78:1057–63. 10.1001/jamaneurol.2021.2403 34338724 PMC8329793

[58] Daneshvar DH Mez J Alosco ML Baucom ZH Mahar I Baugh CM. Incidence of and mortality from amyotrophic lateral sclerosis in national football league athletes. JAMA Netw Open. (2021) 4:e2138801. 10.1001/jamanetworkopen.2021.38801 34910152 PMC8674746

[59] Green-McKenzie J. Commentary for the then and now forum: the healthy worker effect. J Occup Environ Med. (2017) 59:335–46. 10.1097/JOM.0000000000000979 28267105

[60] Walt GS Burris HM Brady CB Spencer KR Alvarez VE Huber BR. Chronic traumatic encephalopathy within an amyotrophic lateral sclerosis brain bank cohort. J Neuropathol Exp Neurol. (2018) 77:1091–100. 10.1093/jnen/nly092 30299493 PMC6927868

[61] Azad TD Li A Pendharkar AV Veeravagu A Grant GA. Junior seau: an illustrative case of chronic traumatic encephalopathy and update on chronic sports-related head injury. World Neurosurg. (2016) 86:515.e11–6. 10.1016/j.wneu.2015.10.032 26493714

[62] Omalu BI Hamilton RL Kamboh MI DeKosky ST Bailes J. Chronic traumatic encephalopathy (cte) in a national football league player: case report and emerging medicolegal practice questions. J Forensic Nurs. (2010) 6:40–6. 10.1111/j.1939-3938.2009.01064.x 20201914

[63] Mantyh WG Spina S Lee A Iaccarino L Soleimani-Meigooni D Tsoy E. Tau positron emission tomographic findings in a former US football player with pathologically confirmed chronic traumatic encephalopathy. JAMA Neurol. (2020) 77:517–21. 10.1001/jamaneurol.2019.4509 31904765 PMC6990867

[64] Hazrati LN Tartaglia MC Diamandis P Davis KD Green RE Wennberg R. Absence of chronic traumatic encephalopathy in retired football players with multiple concussions and neurological symptomatology. Front Hum Neurosci. (2013) 7:222. 10.3389/fnhum.2013.00222 23745112 PMC3662898

[65] Omalu BI Fitzsimmons RP Hammers J Bailes J. Chronic traumatic encephalopathy in a professional American wrestler. J Forensic Nurs. (2010) 6:130–6. 10.1111/j.1939-3938.2010.01078.x 21175533

[66] Arena JD Johnson VE Lee EB Gibbons GS Smith DH Trojanowski JQ. Astroglial tau pathology alone preferentially concentrates at sulcal depths in chronic traumatic encephalopathy neuropathologic change. Brain Commun. (2020) 2:fcaa210. 10.1093/braincomms/fcaa210 33426528 PMC7784042

[67] Ling H Holton JL Shaw K Davey K Lashley T Revesz T. Histological evidence of chronic traumatic encephalopathy in a large series of neurodegenerative diseases. Acta Neuropathol. (2015) 130:891–3. 10.1007/s00401-015-1496-y 26497674

[68] Ling H Kara E Revesz T Lees AJ Plant GT Martino D. Concomitant progressive supranuclear palsy and chronic traumatic encephalopathy in a boxer. Acta Neuropathol Commun. (2014) 2:24. 10.1186/2051-5960-2-24 24559032 PMC3996066

[69] Doherty CP O'Keefe E Wallace E Loftus T Keaney J Kealy J. Blood-brain barrier dysfunction as a hallmark pathology in chronic traumatic encephalopathy. J Neuropathol Exp Neurol. (2016) 75:656–62. 10.1093/jnen/nlw036 27245243 PMC4913433

[70] Buckland ME Sy J Szentmariay I Kullen A Lee M Harding A. Chronic traumatic encephalopathy in two former Australian National Rugby League players. Acta Neuropathol Commun. (2019) 7:97. 10.1186/s40478-019-0751-1 31242928 PMC6595631

[71] Pearce AJ Sy J Lee M Harding A Mobbs R Batchelor J. Chronic traumatic encephalopathy in a former Australian rules football player diagnosed with Alzheimer's disease. Acta Neuropathol Commun. (2020) 8:23. 10.1186/s40478-020-0895-z 32098626 PMC7043040

[72] Lee K Kim SI Lee Y Won JK Park SH. An autopsy proven child onset chronic traumatic encephalopathy. Exp Neurobiol. (2017) 26:172–7. 10.5607/en.2017.26.3.172 28680303 PMC5491586

[73] Alosco ML Cherry JD Huber BR Tripodis Y Baucom Z Kowall NW. Characterizing tau deposition in chronic traumatic encephalopathy (CTE): utility of the McKee CTE staging scheme. Acta Neuropathol. (2020) 140:495–512. 10.1007/s00401-020-02197-9 32778942 PMC7914059

[74] Ameen-Ali KE Bretzin A Lee EB Folkerth R Hazrati LN Iacono D. Detection of astrocytic tau pathology facilitates recognition of chronic traumatic encephalopathy neuropathologic change. Acta Neuropathol Commun. (2022) 10:50. 10.1186/s40478-022-01353-4 35410438 PMC8996534

[75] Postupna N Rose SE Gibbons LE Coleman NM Hellstern LL Ritchie K. The Delayed neuropathological consequences of traumatic brain injury in a community-based sample. Front Neurol. (2021) 12:624696. 10.3389/fneur.2021.624696 33796061 PMC8008107

[76] Wilson L Stewart W. Dams-O'Connor K Diaz-Arrastia R Horton L Menon DK. The chronic and evolving neurological consequences of traumatic brain injury. Lancet Neurol. (2017) 16:813–25. 10.1016/S1474-4422(17)30279-X 28920887 PMC9336016

[77] Geddes JF Vowles GH Nicoll JA Révész T. Neuronal cytoskeletal changes are an early consequence of repetitive head injury. Acta Neuropathol. (1999) 98:171–8. 10.1007/s004010051066 10442557

[78] Hof PR Knabe R Bovier P Bouras C. Neuropathological observations in a case of autism presenting with self-injury behavior. Acta Neuropathol. (1991) 82:321–6. 10.1007/BF00308819 1759563

[79] Williams DJ Tannenberg AE. Dementia pugilistica in an alcoholic achondroplastic dwarf. Pathology. (1996) 28:102–4. 10.1080/00313029600169653 8714284

[80] Omalu BI DeKosky ST Hamilton RL Minster RL Kamboh MI Shakir AM. Chronic traumatic encephalopathy in a national football league player: part II. Neurosurgery. (2006) 59:1086–92. discussion 1092–93. 10.1227/01.NEU.0000245601.69451.27 17143242

[81] McKee AC. The neuropathology of chronic traumatic encephalopathy: the status of the literature. Semin Neurol. (2020) 40:359–69. 10.1055/s-0040-1713632 32712946

[82] McKee AC Stein TD Kiernan PT Alvarez VE. The neuropathology of chronic traumatic encephalopathy. Brain Pathol. (2015) 25:350–64. 10.1111/bpa.12248 25904048 PMC4526170

[83] Braak H Alafuzoff I Arzberger T Kretzschmar H Del Tredici K. Staging of Alzheimer disease-associated neurofibrillary pathology using paraffin sections and immunocytochemistry. Acta Neuropathol. (2006) 112:389–404. 10.1007/s00401-006-0127-z 16906426 PMC3906709

[84] McKee AC Stein TD Crary JF Bieniek KF Cantu RC Kovacs GG. Practical considerations in the diagnosis of mild chronic traumatic encephalopathy and distinction from age-related tau astrogliopathy. J Neuropathol Exp Neurol. (2020) 79:921–4. 10.1093/jnen/nlaa047 32688381 PMC7395709

[85] Mez J Daneshvar DH Alosco ML Alvarez VE Huber BR Stein TD.. Reply to “Chronic traumatic encephalopathy and primary age-related tauopathy”. Ann Neurol. (2020) 88:1052–3. 10.1002/ana.25858 32754941

[86] Daneshvar DH Goldstein LE Kiernan PT Stein TD McKee AC. Post-traumatic neurodegeneration and chronic traumatic encephalopathy. Mol Cell Neurosci. (2015) 66(Pt B):81–90. 10.1016/j.mcn.2015.03.007 25758552

[87] Mckee AC Daneshvar DH. The neuropathology of traumatic brain injury. Handb Clin Neurol. (2015) 127:45–66. 10.1016/B978-0-444-52892-6.00004-0 25702209 PMC4694720

[88] Stern RA Daneshvar DH Baugh CM Seichepine DR Montenigro PH Riley DO. Clinical presentation of chronic traumatic encephalopathy. Neurology. (2013) 81:1122–9. 10.1212/WNL.0b013e3182a55f7f 23966253 PMC3795597

[89] Daneshvar DH Riley DO Nowinski CJ McKee AC Stern RA Cantu RC. Long-term consequences: effects on normal development profile after concussion. Phys Med Rehabil Clin N Am. (2011) 22:683–700. 10.1016/j.pmr.2011.08.009 22050943 PMC3208826

[90] Stern RA Riley DO Daneshvar DH Nowinski CJ Cantu RC McKee AC. Long-term consequences of repetitive brain trauma: chronic traumatic encephalopathy. PM R. (2011) 3(10 Suppl 2):S460–7. 10.1016/j.pmrj.2011.08.008 22035690

[91] NFHS. 2018-19 High School Athletics Participation Survey. High School Participation Survey Archive. (2019). Available online at: https://www.nfhs.org/media/1020412/2018-19_participation_survey.pdf (accessed April 29, 2022).

[92] Alosco ML Stein TD Tripodis Y Chua AS Kowall NW Huber BR. Association of white matter rarefaction, arteriolosclerosis, and tau with dementia in chronic traumatic encephalopathy. JAMA Neurol. (2019) 76:1298–308. 10.1001/jamaneurol.2019.2244 31380975 PMC6686769

[93] Alosco ML Tripodis Y Rowland B Chua AS Liao H Martin B. A magnetic resonance spectroscopy investigation in symptomatic former NFL players. Brain Imaging Behav. (2020) 14:1419–29. 10.1007/s11682-019-00060-4 30848432 PMC6994233

[94] Alosco ML Tripodis Y Fritts NG Heslegrave A Baugh CM Conneely S. Cerebrospinal fluid tau, Aβ and sTREM2 in former national football league players: modeling the relationship between repetitive head impacts, microglial activation, and neurodegeneration. Alzheimers Dement. (2018) 14:1159–70. 10.1016/j.jalz.2018.05.004 30049650 PMC6131058

[95] Kochsiek J O'Donnell LJ Zhang F Bonke EM Sollmann N Tripodis Y. Exposure to repetitive head impacts is associated with corpus callosum microstructure and plasma total tau in former professional American football players. J Magn Reson Imaging. (2021) 54:1819–29. 10.1002/jmri.27774 34137112 PMC8633029

[96] Victoroff J. Traumatic encephalopathy: Review and provisional research diagnostic criteria. NeuroRehabilitation. (2013) 32:211–24. 10.3233/NRE-130839 23535783

[97] Montenigro PH Baugh CM Daneshvar DH Mez J Budson AE Au R. Clinical subtypes of chronic traumatic encephalopathy: literature review and proposed research diagnostic criteria for traumatic encephalopathy syndrome. Alzheimers Res Ther. (2014) 6:68. 10.1186/s13195-014-0068-z 25580160 PMC4288217

[98] Reams N Eckner JT Almeida AA Aagesen AL Giordani B Paulson H. A clinical approach to the diagnosis of traumatic encephalopathy syndrome: a review. JAMA Neurol. (2016) 73:743–9. 10.1001/jamaneurol.2015.5015 27111824 PMC4922002

[99] Katz DI Bernick C Dodick DW Mez J Mariani ML Adler CH. National Institute of Neurological disorders and stroke consensus diagnostic criteria for traumatic encephalopathy syndrome. Neurology. (2021) 96:848–63. 10.1212/WNL.0000000000011850 33722990 PMC8166432

[100] Cullum CM LoBue C. Defining traumatic encephalopathy syndrome — advances and challenges. Nat Rev Neurol. (2021) 17:331–2. 10.1038/s41582-021-00500-0 33947995

[101] Montenigro PH Alosco ML Martin BM Daneshvar DH Mez J Chaisson CE. Cumulative head impact exposure predicts later-life depression, apathy, executive dysfunction, and cognitive impairment in former high school and college football players. J Neurotrauma. (2017) 34:328–40. 10.1089/neu.2016.4413 27029716 PMC5220530

[102] Stamm JM Bourlas AP Baugh CM Fritts NG Daneshvar DH Martin BM. Age of first exposure to football and later-life cognitive impairment in former NFL players. Neurology. (2015) 84:1114–20. 10.1212/WNL.0000000000001358 25632088 PMC4371403

[103] Alosco ML Tripodis Y Koerte IK Jackson JD Chua AS Mariani M. Interactive effects of racial identity and repetitive head impacts on cognitive function, structural MRI-derived volumetric measures, and cerebrospinal fluid tau and Aβ. Front Hum Neurosci. (2019) 13:440. 10.3389/fnhum.2019.00440 31920598 PMC6933867

[104] Adams JW Alosco ML Mez J Alvarez VE Huber BR Tripodis Y. Association of probable REM sleep behavior disorder with pathology and years of contact sports play in chronic traumatic encephalopathy. Acta Neuropathol. (2020) 140:851–62. 10.1007/s00401-020-02206-x 32939646 PMC7669574

[105] Critchley M. Medical aspects of boxing, particularly from a neurological standpoint. Br Med J. (1957) 1:357–62. 10.1136/bmj.1.5015.357 13396257 PMC1974382

[106] Unterharnscheidt F. About boxing: review of historical and medical aspects. Tex Rep Biol Med. (1970) 28:421–95. 4995464

[107] LeClair J Weuve J Fox M Mez J Alosco ML Nowinski CJ. Selection bias analysis supports dose-response relationship between level of American football play and chronic traumatic encephalopathy diagnosis. Am J Epidemiol. (2022). [Epub ahead of print]. 10.1093/aje/kwac075 PMC9989358 35434739

[108] Abdolmohammadi B Uretsky M Nair E Saltiel N Nicks R Daneshvar DH. Duration of ice hockey play and chronic traumatic encephalopathy risk. In: American Academy of Neurology 74th Annual Meeting. (2022).

[109] Kondo A Shahpasand K Mannix R Qiu J Moncaster J Chen CH. Antibody against early driver of neurodegeneration cis P-tau blocks brain injury and tauopathy. Nature. (2015) 523:431–6. 10.1038/nature14658 26176913 PMC4718588

[110] Bakhtiarydavijani A Khalid G Murphy MA Johnson KL Peterson LE Jones M. A mesoscale finite element modeling approach for understanding brain morphology and material heterogeneity effects in chronic traumatic encephalopathy. Comput Methods Biomech Biomed Engin. (2021) 24:1169–83. 10.1080/10255842.2020.1867851 33635182

[111] Zimmerman KA Kim J Karton C Lochhead L Sharp DJ Hoshizaki T. Player position in American football influences the magnitude of mechanical strains produced in the location of chronic traumatic encephalopathy pathology: a computational modelling study. J Biomech. (2021) 118:110256. 10.1016/j.jbiomech.2021.110256 33545573 PMC7612336

[112] Kerwin J Yücesoy A Vidhate S Dávila-Montero BM Van Orman JL Pence TJ. Sulcal cavitation in linear head acceleration: possible correlation with chronic traumatic encephalopathy. Front Neurol. (2022) 13:832370. 10.3389/fneur.2022.832370 35295830 PMC8918564

[113] Noël L Kuhl E. Modeling neurodegeneration in chronic traumatic encephalopathy using gradient damage models. Comput Mech. (2019) 64:1375–87. 10.1007/s00466-019-01717-z

[114] Ghajari M Hellyer PJ Sharp DJ. Computational modelling of traumatic brain injury predicts the location of chronic traumatic encephalopathy pathology. Brain. (2017) 140:333–43. 10.1093/brain/aww317 28043957 PMC5278309

[115] Morrison B Cater HL Wang CCB Thomas FC Hung CT Ateshian GA. A Tissue level tolerance criterion for living brain developed with an *in vitro* model of traumatic mechanical loading. Stapp Car Crash J. (2003) 47:93–105. 10.4271/2003-22-0006 17096246

[116] Ackermans NL Varghese M Williams TM Grimaldi N Selmanovic E Alipour A.. Evidence of traumatic brain injury in headbutting bovids. Acta Neuropathol. (2022) 144:5–26. 10.1007/s00401-022-02427-2 35579705 PMC9217783

[117] Parascandola M Weed DL Dasgupta A. Two Surgeon General's reports on smoking and cancer: a historical investigation of the practice of causal inference. Emerg Themes Epidemiol. (2006) 3:1. 10.1186/1742-7622-3-1 16403213 PMC1343554

[118] Brainard LL Beckwith JG Chu JJ Crisco JJ Mcallister TW Duhaime AC. Gender differences in head impacts sustained by collegiate ice hockey players. Med Sci Sports Exerc. (2012) 44:297–304. 10.1249/MSS.0b013e31822b0ab4 21716150 PMC3694342

[119] de la Cretaz B. More Girls Are Playing Football. Is That Progress? The New York Times. (2018). Available online at: https://www.nytimes.com/2018/02/02/well/family/football-girls-concussions.html (accessed May 6, 2022).

[120] Bernstein L. Flag football: It's the girls' turn to play. Washington Post. (2011). Available online at: https://www.washingtonpost.com/lifestyle/wellness/flag-football-its-the-girls-turn-to-play/2011/11/10/gIQAFpzSON_story.html (accessed May 6, 2022).

[121] Lempke LB Johnson RS Le RK Anderson MN Schmidt JD Lynall RC. Head impact biomechanics in youth flag football: a prospective cohort study. Am J Sports Med. (2021) 49:2817–26. 10.1177/03635465211026643 34264780

[122] Davis GA Castellani RJ McCrory P. Neurodegeneration and sport. Neurosurgery. (2015) 76:643–55. 10.1227/NEU.0000000000000722 25988925

[123] Kovacs GG Horvath MC Majtenyi K Lutz MI Hurd YL Keller E. Heroin abuse exaggerates age-related deposition of hyperphosphorylated tau and p62-positive inclusions. Neurobiol Aging. (2015) 36:3100–7. 10.1016/j.neurobiolaging.2015.07.018 26254956 PMC4609594

[124] Abdolmohammadi B Dupre A Evers L Mez J. Genetics of chronic traumatic encephalopathy. Semin Neurol. (2020) 40:420–9. 10.1055/s-0040-1713631 32712945

[125] Nowinski CJ Cantu RC. Who will protect the brains of college football players? JAMA Neurol. (2021) 78:273–4. 10.1001/jamaneurol.2020.4740 33523092

[126] Baird LC Newman CB Volk H Svinth JR Conklin J Levy ML. Mortality resulting from head injury in professional boxing. Neurosurgery. (2010) 67:E519–20. Corrected and republished in: *Neurosurgery*. (2010) 67:1444–50. discussion 1450. 10.1227/NEU.0b013e3181e5e2cd 20644386

[127] Goldstein LE McKee AC Stanton PK. Considerations for animal models of blast-related traumatic brain injury and chronic traumatic encephalopathy. Alzheimers Res Ther. (2014) 6:64. 10.1186/s13195-014-0064-3 25478023 PMC4255537

[128] van der Staay FJ Arndt SS Nordquist RE. Evaluation of animal models of neurobehavioral disorders. Behav Brain Funct. (2009) 5:11. 10.1186/1744-9081-5-11 19243583 PMC2669803

[129] Massoud TF Hademenos GJ Young WL Gao E Pile-Spellman J Viñuela F. Principles and philosophy of modeling in biomedical research. FASEB J. (1998) 12:275–85. 10.1096/fasebj.12.03.275 9580086

[130] Huang L Coats JS Mohd-Yusof A Yin Y Assaad S Muellner MJ. Tissue vulnerability is increased following repetitive mild traumatic brain injury in the rat. Brain Res. (2013) 1499:109–20. 10.1016/j.brainres.2012.12.038 23276495

[131] Longhi L Saatman KE Fujimoto S Raghupathi R Meaney DF Davis J. Temporal window of vulnerability to repetitive experimental concussive brain injury. Neurosurgery. (2005) 56:364–74. discussion 364-374. 10.1227/01.NEU.0000149008.73513.44 15670384

[132] Raghupathi R Mehr MF Helfaer MA Margulies SS. Traumatic axonal injury is exacerbated following repetitive closed head injury in the neonatal pig. J Neurotrauma. (2004) 21:307–16. 10.1089/089771504322972095 15115605

[133] Prins ML Alexander D Giza CC Hovda DA. Repeated mild traumatic brain injury: mechanisms of cerebral vulnerability. J Neurotrauma. (2013) 30:30–8. 10.1089/neu.2012.2399 23025820 PMC4047842

[134] Kulkarni P Morrison TR Cai X Iriah S Simon N Sabrick J. Neuroradiological changes following single or repetitive mild TBI. Front Syst Neurosci. (2019) 13:34. 10.3389/fnsys.2019.00034 31427931 PMC6688741

[135] Petraglia A Plog B Dayawansa S Dashnaw M Czerniecka K Walker C. The pathophysiology underlying repetitive mild traumatic brain injury in a novel mouse model of chronic traumatic encephalopathy. Surg Neurol Int. (2014) 5:184. 10.4103/2152-7806.147566 25593768 PMC4287910

[136] Bittar A Bhatt N Hasan TF Montalbano M Puangmalai N McAllen S. Neurotoxic tau oligomers after single versus repetitive mild traumatic brain injury. Brain Commun. (2019) 1:fcz004. 10.1093/braincomms/fcz004 31608324 PMC6777515

[137] Mouzon BC Bachmeier C Ferro A Ojo JO Crynen G Acker CM. Chronic neuropathological and neurobehavioral changes in a repetitive mild traumatic brain injury model: chronic effects of r-mTBI. Ann Neurol. (2014) 75:241–54. 10.1002/ana.24064 24243523

[138] Wojnarowicz MW Fisher AM Minaeva O Goldstein LE. Considerations for experimental animal models of concussion, traumatic brain injury, and chronic traumatic encephalopathy—these matters matter. Front Neurol. (2017) 8:240. 10.3389/fneur.2017.00240 28620350 PMC5451508

[139] Shultz SR McDonald SJ Vonder Haar C Meconi A Vink R van Donkelaar P. The potential for animal models to provide insight into mild traumatic brain injury: translational challenges and strategies. Neurosci Biobehav Rev. (2017) 76:396–414. 10.1016/j.neubiorev.2016.09.014 27659125

[140] Pearce AJ Rist B Fraser CL Cohen A Maller JJ. Neurophysiological and cognitive impairment following repeated sports concussion injuries in retired professional rugby league players. Brain Inj. (2018) 32:498–505. 10.1080/02699052.2018.1430376 29388850

[141] Pearce AJ Kidgell DJ Tommerdahl MA Frazer AK Rist B Mobbs R. Chronic neurophysiological effects of repeated head trauma in retired Australian male sport athletes. Front Neurol. (2021) 12:633320. 10.3389/fneur.2021.633320 33767661 PMC7985524

[142] Alosco ML Mian AZ Buch K Farris CW Uretsky M Tripodis Y. Structural MRI profiles and tau correlates of atrophy in autopsy-confirmed CTE. Alzheimers Res Ther. (2021) 13:193. 10.1186/s13195-021-00928-y 34876229 PMC8653514

[143] Stern RA Adler CH Chen K Navitsky M Luo J Dodick DW. Tau positron-emission tomography in former National Football League players. N Engl J Med. (2019) 380:1716–25. 10.1056/NEJMoa1900757 30969506 PMC6636818

[144] Li Y Li Y Li X Zhang S Zhao J Zhu X. Head injury as a risk factor for dementia and Alzheimer's disease: a systematic review and meta-analysis of 32 observational studies. PLoS ONE. (2017) 12:e0169650. 10.1371/journal.pone.0169650 28068405 PMC5221805

[145] Livingston G Huntley J Sommerlad A Ames D Ballard C Banerjee S. Dementia prevention, intervention, and care: 2020 report of the Lancet Commission. Lancet. (2020) 396:413–46. 10.1016/S0140-6736(20)30367-6 32738937 PMC7392084

[146] Doll R. Proof of causality: deduction from epidemiological observation. Perspect Biol Med. (2002) 45:499–515. 10.1353/pbm.2002.0067 12388883

[147] Smith DR Behzadnia A Imawana RA Solim MN Goodson ML. Exposure–lag response of smoking prevalence on lung cancer incidence using a distributed lag non-linear model. Sci Rep. (2021) 11:14478. 10.1038/s41598-021-91644-y 34262067 PMC8280159

[148] Lubin JH Caporaso NE. Cigarette smoking and lung cancer: modeling total exposure and intensity. Cancer Epidemiol Biomarkers Prev. (2006) 15:517–23. 10.1158/1055-9965.EPI-05-0863 16537710

[149] Bren L. Frances Oldham Kelsey. FDA medical reviewer leaves her mark on history. FDA Consum. (2001) 35:24–9. 10.1037/e542662006-006 11444245

[150] Eaton M Schenker M Whorton MD Samuels S Perkins C Overstreet J. Seven-year follow-up of workers exposed to 1,2-dibromo-3-chloropropane. J Occup Med. (1986) 28:1145–50. 3097279

[151] Bouchard C. Literacy and hazard communication: ensuring workers understand the information they receive. AAOHN J. (2007) 55:18–25. 10.1177/216507990705500103 17260677

[152] Weingarten MD Lockwood AH Hwo SY Kirschner MW. A protein factor essential for microtubule assembly. Proc Nat Acad Sci USA. (1975) 72:1858–62. 10.1073/pnas.72.5.1858 1057175 PMC432646

[153] Walsh KM. Boxing: regulating a health hazard. J Contemp Health Law Policy. (1995) 11:23.

[154] Westhead R. Father of Steve Montador files second wrongful death lawsuit against NHL. TSN. (2021). Available online at: https://www.tsn.ca/father-of-steve-montador-files-second-wrongful-death-lawsuit-against-nhl-1.1713358 (accessed May 6, 2022).

[155] Emilee. Olympics hit with lawsuit from the family of dead bobsledder. Courthouse News. (2022). Available online at: https://www.courthousenews.com/olympics-hit-with-lawsuit-from-the-family-of-dead-bobsledder/ (accessed May 6, 2022).

[156] Mazurek M. Former Notre Dame football player sues school, NCAA for negligence in concussion education. South Bend Tribune. (2021). Available online at: https://www.southbendtribune.com/story/news/2021/09/16/notre-dame-football-richard-morrison-ncaa-lawsuit-school-concussions-cte/8364665002/ (accessed May 6, 2022).

[157] Aylwin M. No fix in sight as rugby edges closer to harsh truth hardwired into collision sports. The Guardian. (2021). Available online at: https://www.theguardian.com/sport/blog/2021/dec/12/no-fix-in-sight-as-rugby-edges-closer-to-harsh-truth-hardwired-into-collision-sports (accessed May 6, 2022).

[158] Belson KNFL. Agrees to Settle Concussion Suit for $765 Million. The New York Times. (2013). Available online at: https://www.nytimes.com/2013/08/30/sports/football/judge-announces-settlement-in-nfl-concussion-suit.html (accessed May 6, 2022).

[159] Belson K. NFL Official Affirms Link Between Playing Football and CTE. The New York Times. (2016). Available online at: https://www.nytimes.com/2016/03/15/sports/football/nfl-official-affirms-link-with-cte.html (accessed May 6, 2022).

[160] The Associated Press. NFL agrees to end race-based brain testing in $1B settlement on concussions. NPR. (2021). Available online at: https://www.npr.org/2021/10/20/1047793751/nfl-concussion-settlement-race-norming-cte (accessed May 6, 2022).

[161] Casper ST Bachynski KE Buckland ME Comrie D Gandy S Gates J. Toward complete, candid, and unbiased international consensus statements on concussion in sport. J Law Med Ethics. (2021) 49:372–7. 10.1017/jme.2021.56 34665101 PMC8941977

[162] Allingham, Jeremy. Brain Trust. CBC News. (2020). Available online at: https://newsinteractives.cbc.ca/longform/brain-trust (accessed May 3, 2022).

[163] Brand KP Finkel AM. A decision-analytic approach to addressing the evidence about football and chronic traumatic encephalopathy. Semin Neurol. (2020) 40:450–60. 10.1055/s-0039-1688484 31311037

